# Centering Equity During Health Technology Innovation: Scoping Review of Methods and Research Adjustments to Promote Inclusive Coproduction

**DOI:** 10.2196/89596

**Published:** 2026-07-03

**Authors:** Kara Burns, Carrie Van Rensburg, Shoshana Bloom, Cleva Villanueva, Amio Matenga-Ikihele, Ngaree Blow, Antonela Vogranic, Elizabeth M Crone, Clea Du Toit, Maya G Panniset, Mahima Kalla, Noor El-Dassouki, Sreshta Sheri, Syed Mustafa Ali, Lama Nazer, Lindsay A Stevens, Hasan Ferdous, Husain Salilul Akareem, Raima Lohani, Cecily Gilbert, Bronwen Merner

**Affiliations:** 1 Centre for Digital Transformation of Health, The University of Melbourne, 700 Swanston Street, Carlton, Victoria, 3050, Australia, 61 0414294967; 2 Equiti Health UK, London, United Kingdom; 3 Instituto Politécnico Nacional, Mexico City, Mexico City, Mexico; 4 Moana Connect, Auckland, New Zealand; 5 Faculty of Medical and Health Science, The University of Auckland, Auckland, New Zealand; 6 Melbourne Medical School, The University of Melbourne, Parkville, Victoria, Australia; 7 Peter MacCallum Cancer Centre, Melbourne, Victoria, Australia; 8 Digital Health Validation Lab, University of Glasgow, Glasgow, Scotland, United Kingdom; 9 Centre for Digital Therapeutics, University Health Network, Toronto, ON, Canada; 10 Centre for Health Informatics, University of Manchester, Manchester, England, United Kingdom; 11 Department of Pharmacy, King Hussein Cancer Center, Amman, Jordan; 12 School of Medicine, Stanford University, Stanford, CA, United States; 13 Macquarie University Innovation, Strategy and Entrepreneurship (ISE) Research Centre, Macquarie Business School, Sydney, Australia; 14 Centre for Health Equity, The University of Melbourne, Carlton, Victoria, Australia

**Keywords:** equity, inclusion, digital health technology, priority populations, methods, mobile phone

## Abstract

**Background:**

Digital health has the potential to mitigate health inequity for priority populations who are underserved or marginalized by the health system. However, there is a lack of practical guidance on how to include priority communities in the coproduction of digital health technologies, particularly across the entire lifecycle of innovation, including research, development, and evaluation.

**Objective:**

The aim of this scoping review was to systematically identify and assess published methods used during digital health innovation to promote equitable inclusion of priority communities at every stage of the Centre for eHealth Research roadmap for digital health technologies.

**Methods:**

This review was based on the Arksey and O’Malley framework for scoping reviews. A 6-stage framework was used to execute the review. To increase the trustworthiness of the findings, an expert advisory group was consulted, and their feedback incorporated into the final manuscript. The Participant, Concept, and Context framework was used to structure the inclusion criteria.

**Results:**

The review identified a total of 106 articles, 58 methods, 4 approaches, and 17 research adjustments used to coproduce digital health technologies with priority communities. Common methods across multiple stages included interviews, focus groups, surveys, and workshops; however, the most accessible way to make equity a practical reality during health technology innovation is to appoint a priority population community advisor, or advisory group, from project inception to project closure. Visual and creative methods like photovoice, home tours, and body-mapping were also used, often by priority population researchers themselves. Research adjustments that promote patient safety and comfort, enhanced literacy, peer-support, and recognize sociocultural and demographic considerations have been used to increase the inclusion of priority populations during digital health innovation.

**Conclusions:**

Embedding equity is possible using the practical methods and research adjustments identified to promote inclusive coproduction. Professionals working across health care, health informatics, research, digital health, and technology development can use these findings to center digital health equity during technology innovation. This research also recognizes that coproduction must draw on epistemological frameworks, or ways of thinking, which support Indigenous and other priority population knowledge systems. A solely Western lens risks reinforcing structural barriers and overlooking essential knowledge, as demonstrated by this review when the search strategy missed key scholarly works by priority population authors themselves.

## Introduction

The United Nations’ Sustainable Development Goals advocate for a healthy and prosperous planet, where no one is left behind [1]. However, health care systems across the world continue to fall short in creating equitable opportunities for everyone to achieve optimal health. Tackling health inequities to achieve these goals remains a challenge for global health systems. Digital health technologies (DHTs)—encompassing telehealth, electronic health records, mobile devices and apps, wearables, and artificial intelligence (AI)—present a scalable opportunity to advance health equity by addressing health service accessibility, availability, and capacity concerns [2]. Worldwide, the COVID-19 pandemic accelerated the integration of DHTs across health care, research, government, and industry [3]. However, this digital shift may risk exacerbating poorer health outcomes for some groups, especially those who are already structurally vulnerable [4]. Evidence shows these communities are not able to access the technology at the same rates as people from high-income, non marginalized communities [5]. Thus, mitigating inequity *during* technological innovation is critical to prevent further disadvantage for these priority communities.

Priority communities refer to those who require intentional focus at the system level to ensure fair and equitable allocation of resources, due to historical and ongoing experiences of oppressive policies and systemic marginalization [6]. Such communities may be defined on the basis of social determinants of health, such as socioeconomic status, race, ethnicity, disability, gender, sexuality, or other demographic factors [7]. The use of the term “priority community” emphasizes a strengths-based approach that focuses on the need for investment and resources to promote equity, ensuring the community is prioritized by the health system, rather than being made vulnerable by it [6]. This review focuses on the coproduction of DHTs with priority communities to mitigate inequity.

There is a lack of practical guidance on how to include priority communities in the coproduction of DHTs, particularly across the entire lifecycle of research and development. Previous reviews have focused on frameworks to conceptualize equity on individual, interpersonal, and societal levels without considering technology codevelopment [8-10]; others have generated schemas to address the digital determinants of equity alongside the social determinants of health [11,12] or cataloged barriers and facilitators to advancing digital health equity [13,14]. Some have explored equitable coproduction with a lens on 1 topic [15] or on 1 stage of development (eg, codesign) [16-19].

While frameworks serve to guide principle-based action and develop a common agenda, some scholars are now turning their attention to the application of practical guidance, methods, and approaches [20]. Thus, the aim of the scoping review was to systematically identify and assess published methods and approaches used during digital health innovation to promote equitable inclusion of priority communities at every stage of the Centre for eHealth Research (CeHRes) roadmap, from conceptualization to evaluation [21,22]. The CeHRes roadmap is a holistic framework used to guide the research, development, implementation, and evaluation of digital health technologies. Developed by the Center for eHealth and Wellbeing Research, it ensures that digital health interventions are useful, usable, and embedded within health care contexts and integrates ideas from human-centered design, participatory development, persuasive technology, behavioral science, and business modeling to ensure that digital health solutions fit the needs of users, organizations, and health systems [21,22].

The five stages of the CeHRes roadmap are described below:

1. Contextual inquiry: This stage involves gathering information from intended users and relevant stakeholders about the nature of the problem and potential solutions.
2. Value specification: This stage involves determining and ranking the social, economic, medical, and behavioral values of key stakeholders. From this process, the most favorable solutions are identified as well as the user and organizational requirements to achieve them.
3. Design: This stage involves developing prototypes that align with the values and user requirements.
4. Operationalization: This stage involves the implementation of the technology into practice. Operationalization may include enabling activities, training, education, and deploying the technology into practice.
5. Summative evaluation: This stage refers to the actual uptake of the technology in practice, and its clinical, organizational, and behavioral impacts.

We sought to overlay this guidance across the roadmap, explore any evidence gaps, and discuss the implications of these issues for professionals working across health care, health informatics, research, digital health, and technology development.

Initially, this review was guided by three review questions; however, as the research progressed, a fourth question was identified:

1. What methods and approaches are used when involving priority communities in the development of DHTs?
2. Where are these methods and approaches located along the CeHRes roadmap?
3. How is the acceptability of these methods and approaches described by the priority community or measured by researchers?
4. How were the methods and approaches adjusted for coproduction with the priority community?

To answer these questions, we developed and executed a search, charting, and synthesis strategy as described in the Methods section.

## Methods

### Review Strategy

This review was based on the Arksey and O’Malley framework for scoping reviews [23,24] and was informed by contemporary guidance from the Joanna Briggs Institute [25]. The 6-stage framework includes defining research questions, identifying relevant studies, study selection, charting the data, summarizing, and reporting the results. Additionally, to increase the trustworthiness of the findings, an expert advisory group was consulted and their feedback incorporated into the final manuscript.

This review is based on a published protocol [21]. Differences between the protocol and review included the addition of a fourth research question and the change of language from the word “codevelopment” which could specify just the development phase, to the more encompassing “coproduction” which includes all the phases of research and evaluation. Finally, to provide more specificity, the word “methods” was used in lieu of “tools.” We maintained rigor by fulfilling the PRISMA-ScR (Preferred Reporting Items for Systematic Reviews and Meta-Analyses extension for Scoping Reviews) checklist (Checklist 1).

### Identifying Relevant Studies Using Inclusion and Exclusion Criteria

Eligible articles for this scoping review were peer-reviewed papers published in the English language. The Participant, Concept, and Context framework was used to structure the inclusion criteria (Table 1) [26].

**Table 1.** Inclusion and exclusion criteria.

Framework element	Inclusion criteria	Exclusion criteria
Participant	Considered priority communities, as defined by location (eg, rurality), socioeconomic status, race, ethnicity, disability, age, gender, culture, sexual orientation, or other demographic factors.	The study did not consider priority communities.
Concept	Included approaches or methods for improving digital health equity, defined as equitable inclusion in the development of DHTs^a^.	The study did not include approaches or methods to coproduce DHTs with priority communities.
Context	Incorporated DHTs intended to be used by priority communities to manage health or access healthcare services. As articulated by the National Institute for Health and Care Excellence (UK), DHT includes “standalone software and apps that are used to improve health outcomes or to improve how the health and care system runs” [27]. These can include: regulated medical devices classed as software as a medical device software and apps designed to help people to manage their own health and well-being software that is designed to help the health and care system to run more efficiently or to help staff manage their time, staffing or resources apps or software designed to work alongside a medical device	The study did not incorporate DHTs. DHTs described in study was not intended to be used by priority communities to manage health or access health care services.
Type of articles	Peer-reviewed English-language articles containing empirical research (including qualitative, quantitative, and mixed methods designs).	Commentary, editorial, and protocol articles that did not generate empirical evidence were not eligible. Preprints and theses were excluded. Literature reviews were not included; however, their bibliographies were hand-searched to identify relevant studies.

a DHT: digital health technology.

### Search Strategy

The search strategy aimed to locate published articles and was developed in consultation with a professional librarian (CG). An initial limited search of MEDLINE and ACM Digital Library was undertaken to identify articles on the topic, from both the health and information technology literature. The text words contained in the titles and abstracts of relevant articles, and the index terms used to describe the articles, were used to develop a full search strategy for the targeted databases (Multimedia Appendix 1).

The search strategy, including all identified keywords and index terms, was adapted for each included database or information source. In total, 5 databases (Cochrane Library, Medline ALL on Ovid, CINAHL on EBSCOhost, PsycInfo on Ovid, and Web of Science) were searched from 2010 to December 2023. This period was selected as it coincided with the introduction of smartphones and extended through COVID-19 when digital technology was widely implemented into the community. The reference lists included articles, and any identified systematic reviews were hand searched to ensure completeness. Given that digital health inequities affect priority communities across high-income and low- and middle-income countries, articles from all countries were eligible for inclusion in the review. To improve the feasibility of the review, traditional imaging that has been digitized and data-driven technologies (blockchain and AI-based algorithms) were excluded as these are not routinely used in the delivery of clinical health care services.

### Evidence Selection

Following the search, all identified citations were collated and uploaded into the Covidence software (Veritas Health Innovation) for removal of duplicates and management of the subsequent stages of the review screening and data extraction processes [28]. Dual and independent screening was conducted for 10% of titles and abstracts (CVR, SS, and KB). When agreement between the reviewers was equal to or greater than 80%, single screening was conducted for the remaining yield (CVR). Potentially relevant articles were retrieved in full.

The full text of selected citations was assessed in detail against the inclusion criteria by 2 or more independent reviewers (all authors). The reasons for excluding full-text articles that did not meet the inclusion criteria were recorded and reported. Any disagreements that arose among the reviewers at each stage of the selection process were resolved through discussion, or with an additional reviewer (KB or CVR).

### Data Extraction

Data was extracted from articles by a reviewer (all authors) using a data extraction tool developed and piloted for the project. The data were then checked by a second reviewer (CVR), and any disagreements were addressed by a third reviewer (KB). The data extracted included specific details about the participants, concept, context, study methods, study location (classified using the World Bank Country Classification [29]), and key findings relevant to the review questions and mapped to the digital innovation pathway through the CeHRes roadmap for the development of eHealth technologies (Multimedia Appendix 2).

### Data Analysis and Presentation

Methods and approaches were identified and deductively mapped to the CeHRes roadmap for the development of eHealth technologies. To identify methods that were successfully used with priority populations, we sought to identify any “validation” of method use. Primarily this was through any reference to acceptability of the methods use, from the perspective of priority populations. However, literature also indicated we may find examples of researcher reflexivity, or the practice of critically reflecting on one’s own role, assumptions, and biases, during the innovation process [30]. This data were extracted and presented as a typology. Finally, adjustments to the research or methods to make them more suitable for the priority population were extracted from papers and thematically analyzed using an inductive process.

## Results

### Overview

**Figure 1.**
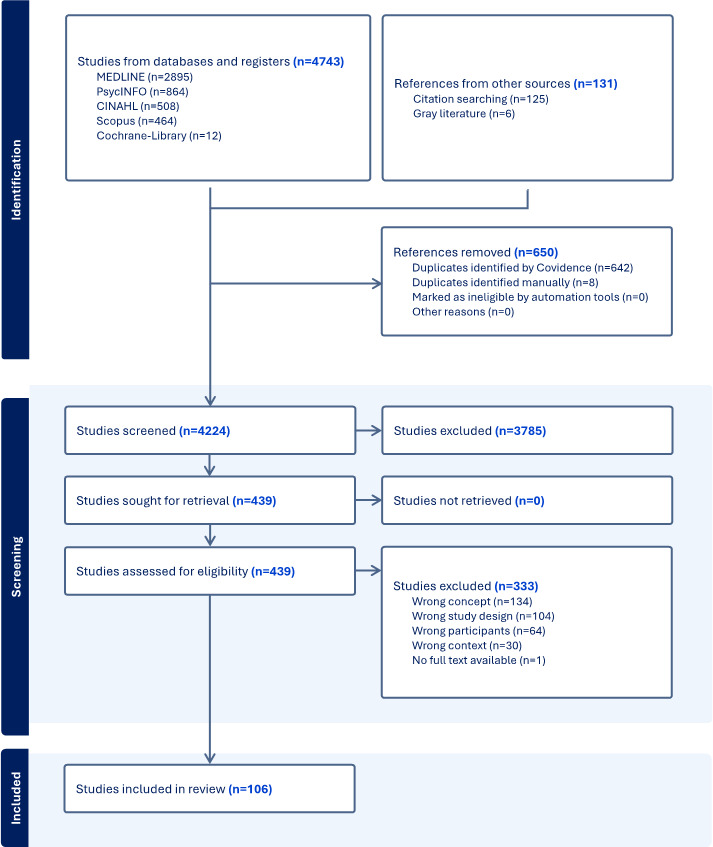
PRISMA (Preferred Reporting Items for Systematic Reviews and Meta-Analyses) flow diagram.

The scoping review identified a total of 106 articles [31-136] that met the inclusion criteria and were included in the final analysis. Our search produced 4743 articles from databases or registers and an additional 131 articles from other sources. Of the total, 650 articles were eliminated after being identified as duplicates. In total, 4224 articles were abstract- and title-screened. Of these, 439 articles were advanced to full-text review, and 106 articles were ultimately included in the scoping review. Figure 1 shows the PRISMA (Preferred Reporting Items for Systematic Reviews and Meta-Analyses) flow diagram, which details the reasons for exclusion.

### Study Characteristics

**Figure 2.**
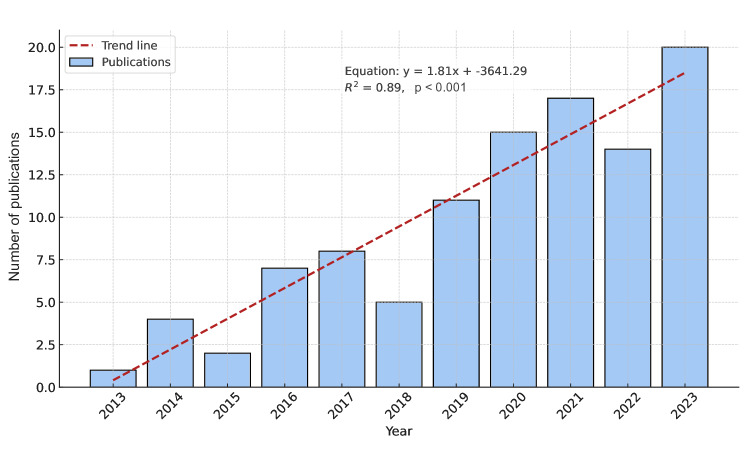
Number of publications per year (2013‐2023).

We extracted and reported key study characteristics summarized in Multimedia Appendix 3 to support analysis of publication trends. As seen in Figure 2, the number of papers exploring digital health equity published per year trended upwards throughout the review period from 1 in 2013 to more than 20 in 2023.

Most papers were from high-income countries (n=93) [31-123], with the United States producing the most studies of any country (n=58) [31,32,35-43,47-52,56-60,62-64,66,68,69,71,73,76,77,79,81,82,84-87,89-91,93,94,98-100,105,111-120], followed by Australia (n=11) [31,32,35-43,47-52,56-60,62-64,66,68,69,71,73,76,77,79,81,82,84-87,89-91,93,94,98-100,105,111-120], the United Kingdom (n=6) [44,65,72,75,102,106], and Canada (n=5) [54,61,74,96,108]. Coproduction with priority populations in low- and middle-income countries was low compared with high-income countries, with only 14 studies across upper-middle-income (n=5) [124-128], lower-middle-income (n=7) [45,129-134], and low-income countries (n=2) [135,136]. Moreover, 1 study conducted research in both a high-income and a lower-middle-income country [45].

### Priority Populations

**Figure 3.**
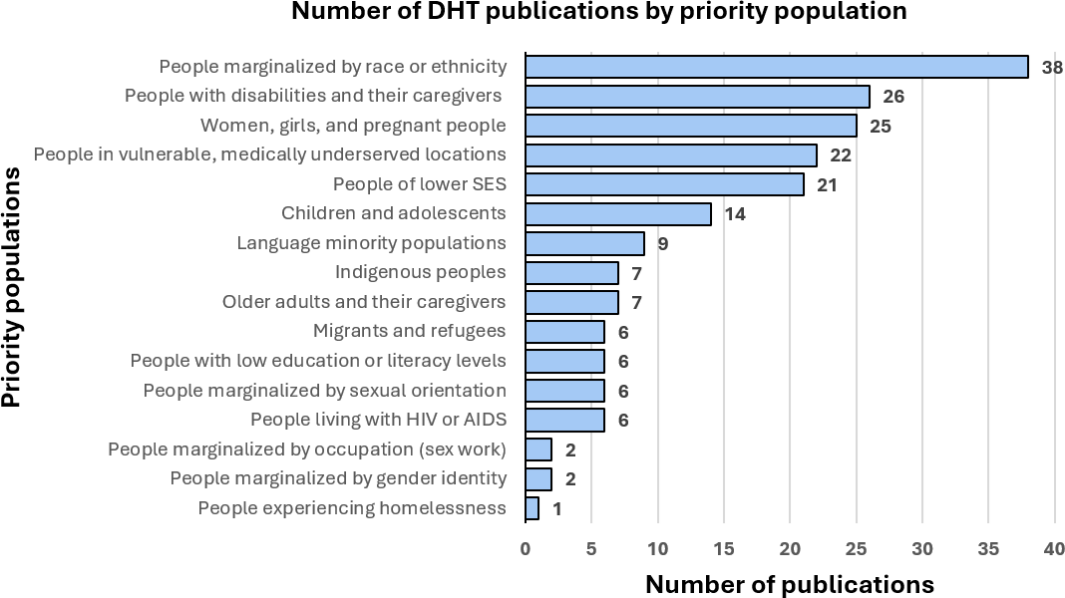
Priority population groups represented across the 106 papers. DHT: digital health technology; SES: socioeconomic status.

Across the 106 articles [31-136] reviewed, 16 priority population groups were involved in the coproduction of digital health technologies at least once (Figure 3). These categories are drawn from the United Nations Human Development Programme Report (2016), and they are analytic aids rather than mutually exclusive groups. Individuals may belong to both categories, and the overlapping identities may result in multiple experiences of marginalization. Recognizing this, we have addressed coproduction of digital health technology with people who may have overlapping identities later in this paper [137].

People marginalized by race or ethnicity were included most often (n=38) [37,40,42,43,46-48,50-52,55,58-60,63-65,68,69,75,80,82,84-87,91,93,94,99,100,102,111-114,116,118], with women, girls, and pregnant people [37,40,42,46,48,50-52,63,64,68,70,79,84,86,87,96,105,115,116,120], people in vulnerable locations [32-34,36,41,45,47,53,56-58,72,73,83,88,89,97,103,107,117,131,136], people with disabilities [35,38,39,44,55,61,65,67,71,74-78,81,83,91,106,108-110,112,113], and people of lower socioeconomic status [47,48,53-55,60,63,65,68,71,79,82,90,91,94,96,100,101,115,122,123] were each the focus in more than 20 articles. People experiencing homelessness [72], people marginalized by occupation [117,131], and people marginalized by gender identity [102,119,126] were represented least, each included within 3 or fewer articles. Some articles focused on coproducing DHTs with participants who held multiple identities (Multimedia Appendix 4). A total of 42 articles [32-36,38,41,44,45,53,54,56,57,61,62,66,67,69,71-73,75,76,78,81,83,88-90,93,95,98,102,103,106,108,109,112,114,117,131,136] focused on 1 specific priority population; however, on average, articles centered participants who held 2 intersecting identities that may have a compound effect on inequitable experiences of and outcomes from using digital health technologies (Range: 1‐4).

### DHTs Typology

Across the 106 papers [31-136], all DHTs being coproduced with priority communities had a consumer-facing component. With an estimated 95 definitions of digital health in the literature and multiple frameworks describing different modalities, classifying DHTs in a succinct schema proved challenging. Initially, we tried classifying DHTs using guidance from the World Health Organization [138], the Digital Therapeutic Alliance [139], and the National Institute for Health and Care Excellence Evidence Standards Framework for Digital Technologies (United Kingdom) [27]. All proved insufficient for defining direct-to-consumer technology classification with in-depth detail.

Based on advice from our international expert advisory committee (refer to Acknowledgments section), we have developed our own classification system for consumer-facing DHTs. For direct-to-consumer DHTs, we focused on the *technology type* or the platforms used to deliver the technology. Key parameters for classification included the *engagement mechanism* or tasks undertaken by users (eg, information, digital comics, and electronic distress monitoring tool), the *user engagement model* describing who used the technology, the *primary function* the technology addressed in the health system, and the *clinical condition or health domain* where the technology was applied. Refer to Multimedia Appendix 5 for the full results and Table 2 for a summary of the *technology type*, *user eng*ag*ement model*, and *primary health function*.

**Table 2.** Classification of direct-to-consumer digital health technologies^a^.

Domain	Description	Papers, n (%)
Technology type		
Mobile apps	Standalone smartphone and tablet apps	54 (50.9)
Web-based platforms	Browser-accessible websites and portals	31 (29.8)
Messaging systems	SMS and text-based interventions	12 (11.3)
Telehealth and virtual care	Real-time remote consultation platforms	10 (9.4)
Wearable and IoT^b^	Wearable devices and IoTs integration	7 (6.6)
Social media	Social networking platforms	2 (1.9)
Automated phone systems	Interactive voice response systems	1 (0.9)
Not specified	—^c^	3 (2.8)
User engagement model		
Self-directed	Patient-initiated, autonomous engagement	58 (54.7)
Self-directed (caregiver)	Caregiver-initiated, autonomous engagement	7 (6.6)
Self-directed (patient and caregiver)	Both patient and caregiver-initiated, autonomous engagement	6 (5.7)
Guided or coached	Professional (nonclinical) support integrated	6 (5.7)
Provider-mediated	Healthcare professional involvement required	27 (25.5)
Peer-supported	Community or peer interaction features	11 (10.4)
Automated	System-initiated reminders and notifications	11 (10.4)
Caregiver-supported	Support provided by caregivers	2 (1.9)
Not specified	—	3 (2.8)
Primary health function		
Health education and literacy	Disease prevention and health maintenance	43 (40.6)
Assessment and screening	Health evaluation and diagnostic support	9 (8.5)
Clinical and disease management	Treatment delivery and adherence	23 (21.7)
Psychological support and well-being	Psychological support	12 (11.3)
Behavioral change support	Lifestyle modification and habit formation	25 (23.6)
Care coordination	Healthcare navigation and service coordination	9 (8.5)
Self-monitoring	Symptom tracking	15 (14.2)
Not specified	—	2 (1.9)

a The percentage of papers does not add up to 100% as some papers included DHTs that combined technology types, use engagement models, and primary health functions.

b IoT: Internet of Things.

c Not applicable.

### Clinical Condition or Health Domain

The most frequently cited health domain was mental health, which appeared 16 (15.1%) times across a range of contexts. This included specific subdomains, such as peripartum depression [120], posttraumatic stress disorder or bipolar disorder [38], anxiety and depression [45,86,87,101,124], and digital services [56,57,74,76,92], as well as general mental health support in relation to parenting, nutrition, chronic illness [63], perinatal health [64], and other well-being services where the condition was not specifically described [80,97]. Next most frequently cited was technology to support people living with HIV, appearing 11 (10.4%) times, highlighting prevention [51], clinic attendance and service provision [66,127], care outcomes for at-risk communities [31,62,105,119,126,128], and disease management [94,131].

Physical activity was the target of 6 (5.7%) interventions, reflecting its role as a key behavioral intervention across health domains [48,103,111,117,121,123]. Technology was also applied to diabetes management in 6 (5.7%) studies, including general management [60,71,82,85] and gestational diabetes management [68,116]. Smoking cessation was also targeted with 5 (4.7%) technologies [59,88,89,114,115]. General health appeared 5 (4.7%) times, typically indicating non–condition-specific or whole-person health promotion approaches [53,90,118,132,134]. Finally, 5 other clinical conditions or health domains had 3 technology applications each, namely, breast cancer or health [37,42,52], prenatal care [40,46,47], managing disability [67,77,78], complex chronic conditions [33,34,75], and multimorbidity [65,69,91].

### Approaches Used When Involving Priority Communities in the Coproduction of DHTs

#### Overview

Four dominant approaches for involving priority communities in the coproduction of DHTs were evident in the reviewed studies, each offering distinct contributions to the innovation of DHTs: participatory design, user-experience testing, community-based participatory research, and ethnography.

#### Participatory Design

The most common approach used by studies was participatory design (also referred to as co-design or user-centered design), which foregrounds the involvement of users throughout the technology development process [36,39,45,47,53,55,60,63-65,67,70,75,79,85,91,92,94,96,97,108,110,115,121,123,124,131,133,136]. Rooted in democratic and inclusive design traditions, these studies engaged diverse stakeholders including patients, carers, and clinicians as active partners in the technology development process. Participatory methods facilitated mutual learning between developers and users, ensuring that technologies aligned with the values, preferences, and lived experiences of intended end users.

#### User-Experience Testing

A second approach evident in the literature focused on user-experience testing. These studies typically involved structured usability tasks, where participants interacted with prototypes or functioning systems under observation [38,41,45,52,73,78,86,92,100,103,105,107-109,113-115,121,134,136]. Participants were often asked to “think aloud” while completing tasks, allowing researchers to identify usability barriers, interface design issues, and cognitive load. This approach provided practical insights into system performance and user satisfaction, informing iterative design improvements.

#### Community-Based Participatory Research

A third group of studies used a community-based participatory research approach, which has emerged from the field of public health where equitable research partnerships between communities and academics are prioritized [33,34,43,48,50,58,69,72,84,111,117]. These studies fostered long-term collaborations with community partners at multiple stages of the research project to increase cultural relevance and support implementation, fostering the sustainability of the project [140].

#### Ethnographic Research

In total, 2 studies adopted ethnographic methods, drawing from anthropology and sociology, to explore the everyday practices, routines, and contexts of users [36,65]. These studies involved immersive in-person work, including participant observation and in-depth interviews, enabling researchers to understand digital health needs and behaviors from an insider’s perspective. Ethnography was often used to surface tacit knowledge, social dynamics, and contextual factors often overlooked in more structured approaches.

### Methods Used When Involving Priority Communities in the Coproduction of DHTs and Their Location Along the CeHRes Roadmap

**Figure 4.**
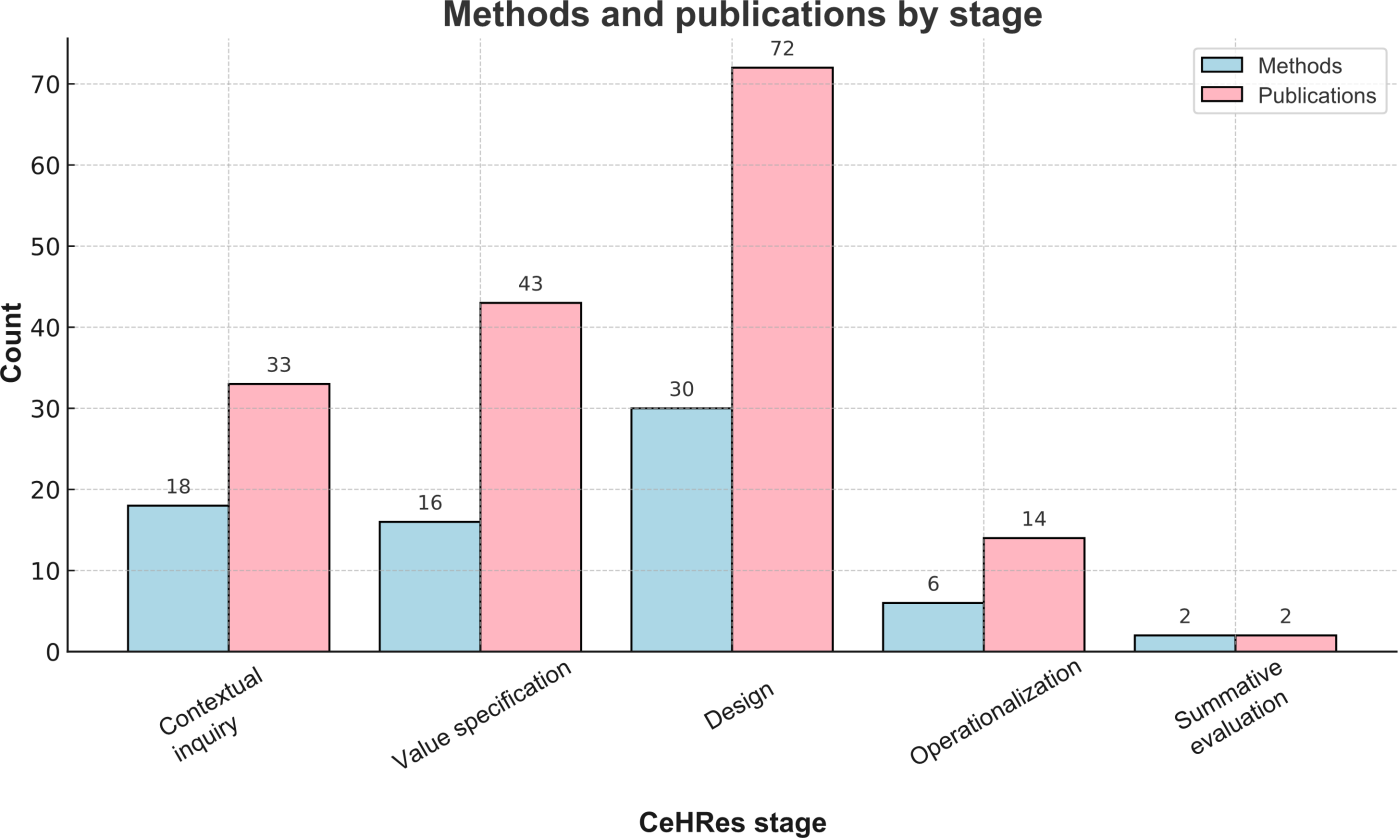
The number of methods and publications at each stage of the CeHRes roadmap. CeHRes: Centre for eHealth Research.

A total of 58 unique research methods were described in 106 articles [31-136] across the 5 stages of the CeHRes roadmap to coproduce DHTs with priority populations. These numbers partially correspond to the number of publications per stage (Figure 4) with multiple methods used in some papers, and several papers and methods used across multiple stages.

**Figure 5.**
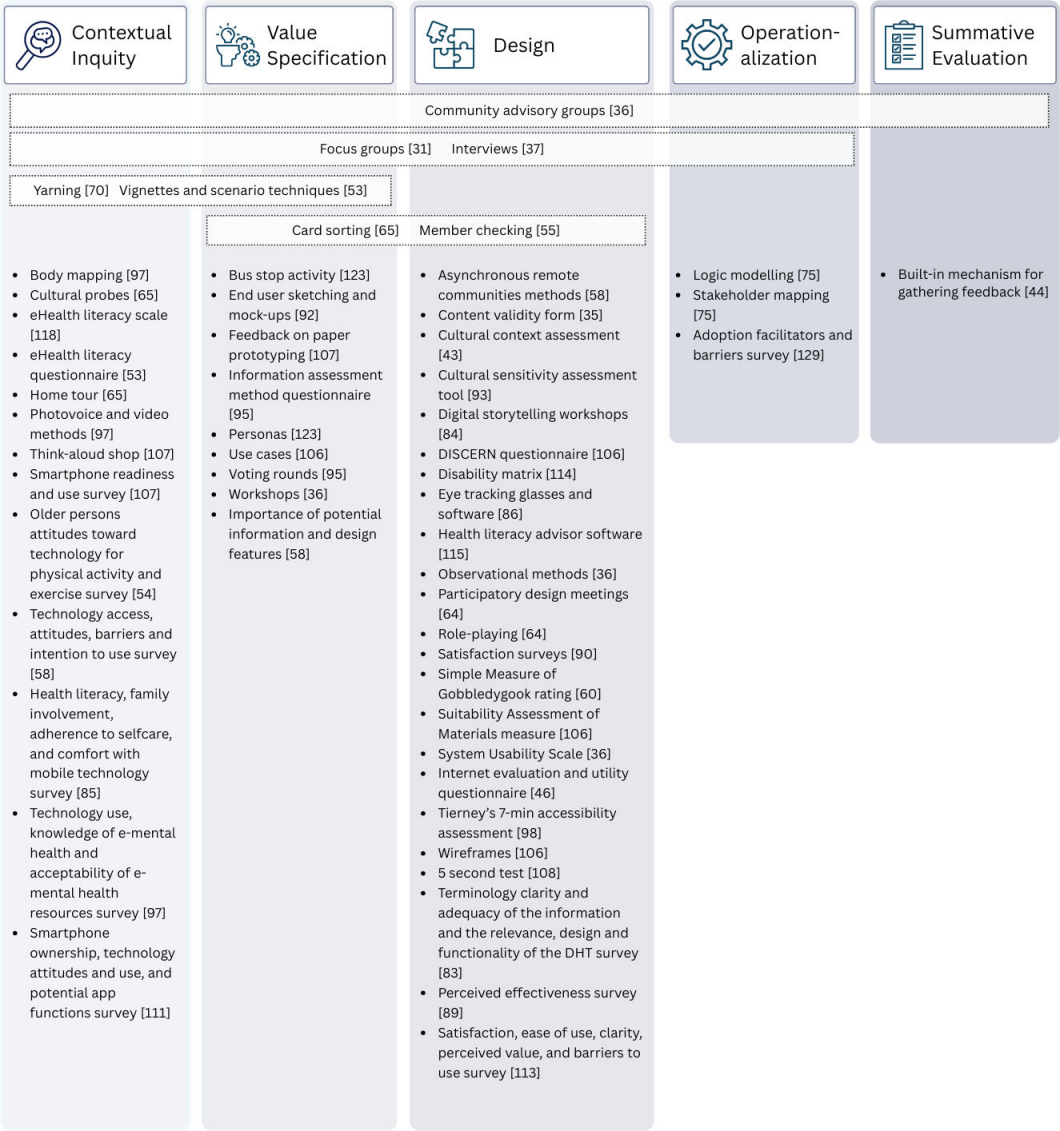
Methods mapped to the stages of the CeHRes roadmap. CeHRes: Centre for eHealth Research.

Most methods were used in the *design* stage (n=30), with *contextual inquiry* second (n=18), and *value specification* third (n=16). *Operationalization* (n=6) and *summative evaluation* (n=2) were poorly represented in the results, with fewer methods than at other stages. Although most methods were present in the *design* stage, *contextual inquiry* had the best ratio of methods to studies, demonstrating that researchers are interested in and have methods to use in the early stage of coproduction before design and development. Refer to Multimedia Appendix 6 and Figure 5 for the full details of methods per stage and their descriptions.

Interviews emerged as the most used method, used in 58/106 (54.7%) studies and across 4 of the 5 stages. Focus groups were used across 4 of the 5 stages and used in 41 (38.7%) of studies. Card sorting [65,91,121], member checking [55,97], yarning [70], and vignette or scenario techniques [53,65,67,97] were used across 2 of the 5 stages. Community advisory groups was the only method used across all stages of the roadmap, demonstrating the flexibility of the approach, although it was only used in 9 studies [35,36,58,66,74,97,111,122,124].

*Contextual inquir*y methods focused on understanding the lives, roles, and needs of stakeholders, as well as the broader sociotechnical environment in which the technology would be deployed. Innovative techniques such as photovoice [97], home tours [65], body mapping [97], and custom-developed or coproduced surveys measuring technology use, attitudes, and barriers were used.

In the *value specification* stage, the emphasis shifted to identifying what mattered most to users and stakeholders. These methods helped prioritize values and translate them into actionable design goals. Techniques included workshops [36,55,84], voting rounds [95], persona development [67,123], use-case scenarios [106], and feedback on paper prototypes [107]. Notably, member checking [55,97] and community advisory input [35,74,124] were used to validate interpretations and ensure alignment with participant expectations.

*Design* methods aim to translate insights into tangible prototypes and assess their appeal, usability, and clarity. These ranged from basic wireframes and mock-ups [106] to more complex testing using eye-tracking software [86,114], accessibility assessments, and validated instruments, such as the System Usability Scale [36,38,41,51,73,94,98,127], DISCERN questionnaire [106], and the Suitability Assessment of Materials [106]. Participatory techniques, such as role-playing [64], digital storytelling workshops [84], and participatory design meetings, ensured user involvement remained central.

For *operationalization*, the focus was on implementation planning and business model development. This phase drew on stakeholder mapping [75], logic modeling [75], and surveys that explored perceived barriers and facilitators to adoption [129], in addition to maintaining continuity with earlier-phase engagement methods like interviews and community advisory groups [58].

Finally, *summative evaluation* assessed real-world uptake and impact, using built-in feedback mechanisms (user engagement data via Google Analytics and a Feedback button on the homepage) [44] and continued engagement with community advisory groups to monitor performance and acceptability.

### The Acceptability of Methods and Approaches Described by the Priority Community or Measured by Researchers

In total, 13 (12.3%) studies reported acceptability of their methods when coproducing with priority populations. Acceptability in this context is defined as any subjective or objective evidence to understand if the method was effective and acceptable for the priority population. A total of 5 distinct techniques were identified, varying in formality and depth, ranging from experience surveys and evaluation via comparison with current best practice [36] to researcher reflexivity [91,104,110] and assessment using a culturally led framework [70].

The most used validation technique was community consultation, where the research methods were reviewed and adjusted by the priority population before the research was conducted. Table 3 outlines each technique, provides examples of how it was applied, and lists the corresponding references. Together, these techniques reflect a diversity of strategies used to assess methodological acceptance in digital health innovation involving priority populations.

**Table 3.** Validation techniques.

Acceptability technique	Examples	References
Assessment using a culturally led framework	The research design and study were guided by and evaluated against criteria set by the Australian National Health and Medical Research Council’s ethical guidelines for research involving Aboriginal and Torres Strait Islander people.	Henson et al [70]
A priori community consultation	Interview schedules were developed in consultation with the project advisory group comprising 8 participants, including 3 young people, 1 parent, and 4 health and education professionals and nongovernmental representatives [124].	Brooks et al [124], Ceasar et al [48], Henson et al [70], Hoque and Sorwar [129], Hynie et al [74], Povey et al [97], Vangeepuram et al [111]
Comparison with best practice.	The codeveloped digital myPath app showed the highest perceived combined usability (mean 81.9, SD 15.2) compared with the current gold standard of distress management for patients with cancer, the paper-based National Comprehensive Cancer Network Distress Thermometer (mean 74.2, SD 15.8).	Aronoff-Spencer et al [36]
Qualitative reflections or quantitative measures of participant experience	During session 3, they used Jamboard to facilitate the discussion “*How do you feel about the workshops now that you have completed them?*” After session three, participants completed a survey about their experience, using a 4-point Likert scale, which included items such as “After completing the digital storytelling workshops, I feel this was worth my time” [84].	Maragh-Bass et al [84], Zapata et al [119]
Researcher reflexivity	“However, we expected more consistency in the feedback on existing materials, as end users are usually more engaged in this type of feedback...it is also possible that the participant’s response bias owing to the interviewer’s demand characteristics contributed to the differences in feedback by health and digital literacy and English proficiency” [91].	Nouri et al [91], Spanhel et al [104], Van Doreen et al [110]

### How Has the Research Team Adjusted the Methods and Approaches for Coproduction With the Priority Community?

Across the 106 papers [31-136], 17 methodological adjustments were implemented to ensure the coproduction process was inclusive, culturally safe, and responsive to participant needs. These were inductively themed into (1) participant safety and comfort, (2) enhancing literacy, (3) peer-support, and (4) social, cultural, and demographic considerations (Table 3).

In total, 40 (37.7%) studies used at least 1 research adjustment. Henson et al [70] used the most adjustments (n=5) to identify how older Aboriginal and Torres Strait Islander women use DHTs to enhance their own health: using yarning rather than interviews or a focus group, taking a strengths-based approach, peer-led recruitment, a peer researcher, and using an Aboriginal project governance group. Enhancing literacy was a key consideration when adjusting for priority population inclusion. Out of the 38 studies, 27 (71%) that used adjustments improved involvement in the coproduction of DHTs by conducting the methods in the language of the priority population (n=25), adapted methods or instructions for low literacy levels (n=6), and/or providing paper alternatives (n=1). Refer to Table 4 for the full results; some studies used multiple methods.

**Table 4.** Research adjustments (participant safety and comfort, enhancing literacy, peer-support, and social, cultural, and demographic considerations).

Adjustment	Examples	References
Participant safety and comfort		
Accessibility consideration	Thinking about room access, options for any special diets, and developing ground rules to ensure everyone was able to participate equally.	Russ et al [102]
Checking in with participants’ comfort level	Use of emotion cards featuring licensed stock images of young adults of color, numbered from 1 to 6, without labels but with clearly portrayed emotions (eg, shyness and excitement) to gauge participants’ comfort levels.	Maragh-Bass et al [84]
Data collection conducted in a location that meets the needs of participants	Focus group conducted at a local church or interviews in a person’s home and a local café.	Calderón et al [125], Ceasar et al [48], Doty et al [58]
Individualized implementation methods	Used a daily phone call strategy to enhance engagement with the app and minimize dropouts during pilot testing, particularly among Spanish-speaking participants and those with limited health and digital literacy	Pathak et al [121]
Steps taken to ensure privacy	To ensure anonymity, the participants were instructed to use a pseudonym for their name on video discussions.	Peng et al [126], Zapata et al [119]
Enhancing literacy		
Adapting methods and instructions for low literacy levels	Interviewers modified the administration of the activity to accommodate participants with limited literacy or communication barriers by providing audiovisual cues, including reading the cards aloud and successively probing for feedback after reading each card	Pathak et al [121], Nouri et al [91], Meijer et al [88], Kang et al [78], Tonkin et al [107], Povey et al [97]
Nondigital alternatives	Produced both online and paper-based versions of the surveys	Swallow et al [106]
Conducted in the language of the priority population or engaged an available interpreter	Researchers who shared the same language background as the participants facilitated the codesign workshops to ensure cultural safety and enable engagement using participants’ first language.	Albright et al [32], Burchert et al [45], Bravo et al [42], CalderÃ³n et al [125], Campbell et al [135], Cerda Diez et al [49], Dobson et al [122], Fontil et al [60], Garvelink et al [61], Handley et al [68], Doty et al [58], Hearn et al [136], Hoque and Sorwar [129], Hutchings et al [75], Hynie et al [74], Kayastha et al [130], Lindegaard et al [80], Mueller et al [133], Nouri et al [91], Petros De Guex et al [94], You et al [128], Mauka et al [131], Pathak et al [121], Petros De Guex et al [94], Povey et al [97]
Peer-support		
Allow accompaniment of a support person	Patients that were accompanied by a caregiver were given the option of (1) using the system themselves or (2) having their caregiver use the system on their behalf.	Hearn et al [136]
Peer-led recruitment	Recruitment of participants by peers (people with similar lived experience). For example, recruitment of female sex worker participants was led by female sex worker peers.	Mauka et al [131], Henson et al [70], You et al [128]
Peer researcher	Researchers with similar lived experience to participants helped facilitate data collection. Peer researchers may also receive research skills training to help build capacity.	Howells et al [72], Povey et al [97], Henson et al [70], Verbiest et al [123]
Social, cultural, and demographic considerations		
Aboriginal project governance	Recognition of the inherent right of Indigenous peoples to self-determination and control over their own research, data, and knowledge through ensuring their priorities and values are reflected in research design, implementation, and outcomes.	Henson et al [70]
A consideration of group dynamics	Focus groups split by gender, age, ethnicity, or sexual orientation.	Bounds et al [41], Burchert et al [45], Dobson et al [122], Rozbroj et al [101], Verbiest et al [123]
Using culturally appropriate research methods	Using culturally appropriate data collection methods. For example, using yarning rather than focus groups with Aboriginal communities.	Henson et al [70]
A consideration of personal beliefs	All meetings began with an opening prayer by church leadership to set an atmosphere of creativeness, inspiration, and togetherness among the attendees.	Brewer et al [43]
Taking a strengths-based approach	Framing the disconnect between the intended health message and the person’s understanding of the meaning resulting from inaccessible language and impractical solutions to center the problem with the content creator, rather than health or digital literacy of the individual.	Henson et al [70]
Use of art and play to engage children and young people	The use of arts activities like drawing, model making, and sculpting with playdough to ascertain preferences for intervention content, format, delivery, and implementation	Brooks et al [124]

## Discussion

### Principal Findings

Multiple systematic, scoping, and narrative reviews have focused attention on the opportunity of digital health innovations to reduce inequity and improve health outcomes for priority populations. Evidence is mounting that coproduced technologies, where users and community partners are consulted throughout the research, development, and evaluation process, are more acceptable to priority populations than those created without them [4,36,53,141,142]. The aim of the scoping review was to systematically identify and assess published methods and approaches used during digital health innovation to promote equitable inclusion of priority communities at every stage of the CeHRes roadmap, from conceptualization to evaluation [21,22]. Existing reviews demonstrating the practical application of equitable methods have largely focused on the design stage of technology development [16-19]. Therefore, we advance and expand the practice of including priority communities in digital health innovation across the whole innovation pathway by cataloging 58 practical methods, 4 approaches, and 17 adjustments. Indeed, for professionals working across health care, health informatics, digital health, and technology development, this review provides practical, implementable strategies to center equity throughout the entire technology innovation pathway [143-145] in Multimedia Appendix 7.

Coproduction of digital health innovations with priority populations increased between 2013 and 2023. This review demonstrates the early-to-mid stages of innovation, particularly the *design* stage, encompassed the most studies and methods. This is concordant with the proliferation of literature on co-designing DHTs [146]. Additionally, we show that both during conceptualization and when specifying value, priority groups can be included, enabling them to be involved before an innovation is designed or developed into a solution. This aligns with recent advice from research funding bodies to create authentic public and patient partnerships from project inception in order to improve research relevance, enhance research impact, strengthen the research process, and increase trust and engagement [147].

Although the CeHRes roadmap originates from Western research traditions, its application does not inherently reinforce inequity when priority communities are meaningfully engaged from the earliest stages of innovation. Involving priority communities from project inception—particularly during contextual inquiry and value specification—allows their knowledge systems, priorities, and lived experiences to shape the development of digital health technologies. In this way, the CeHRes roadmap can function as a flexible analytical structure rather than a prescriptive framework, supporting inclusive coproduction rather than imposing external assumptions.

We also note that fewer studies and methods focused on the *implementation* and *evaluation* stages. Although many digital health implementation papers focus on the clinical outcomes for priority populations, they fail to include these populations in the interpretation of the results. This is a clear gap and should be a focus of future research. Without a situated narrative and an understanding of implementation complexities that influence the equitable uptake, scale, spread, and sustainability of these digital health innovations, there is a risk they will not achieve the proposed equity aims [148].

Encouragingly, all priority populations defined by the United Nations Human Development Programme Report (2016) were featured in at least 1 paper [31,32,35,37,40,42,47,53,61,70,72,78,101,102,131]; however, as most studies were conducted in high-income countries, it is unclear to what extent these methods could be applied to other socioeconomic settings. Even within high-income countries, people experiencing homelessness, people marginalized by occupation (eg, sex workers), people marginalized by gender identity (eg, trans people), and Indigenous peoples were underrepresented. This research supports the use of adjustments to enhance participants’ research experience when coproducing technology. In some of these cases, the presence of multiple intersecting identities led to a greater number of research adjustments. Based on this review, the lack of consideration of intersectionality and how it applied in digital health coproduction is a key concern. Future research should recognize multiple identities and, therefore, multiple disadvantages, using research adjustments to overcome these disadvantages.

Priority populations’ health outcomes are driven by colonization, structural inequality, and ongoing discrimination, not biology [149]. To better understand these results, in the next section, we have reflected on them from an Indigenous Australian perspective through the lens of decolonization.

### Priority Population Perspective

Decolonizing research in digital health requires a critical interrogation of the colonial structures that shape research processes, from topic selection to dissemination, and a deliberate recentering of Indigenous ways of knowing, being, and doing [150]. Decolonizing research involves “evaluating and dismantling and disrupting Western ways of knowing”—in this case the foundations of research practice as it stands [151]. In reviewing studies pertaining to Aboriginal and Torres Strait Islander communities, this scoping review identified multiple adjustments, methods, and approaches, such as Aboriginal project governance, yarning methodologies, peer researchers, strengths-based frameworks, and active community participation. Categorizing these elements proved challenging, as many could be simultaneously understood as an approach, an adjustment, or a method, and were often applied across multiple stages of the CeHRes roadmap. This difficulty in classification reflects the tension between Indigenous research practices, which are relational, iterative, and context-specific, and Western research systems that prioritize standardization, rigid categorization, and linear progression. As noted by Laycock et al [152], Indigenous research methodologies emphasize Indigenous control of the research agenda, alongside respectful processes for consultation and negotiation. These principles resist prescriptive methods and instead demand flexibility to meet local community priorities. However, fitting such approaches into frameworks developed within colonial systems risks reducing them to technical steps rather than honoring them as holistic practices.

There is growing evidence that recentering Indigenous ways of knowing, being, and doing strengthens research quality and relevance for First Nations peoples [153]. This is particularly critical when working with data relating to Aboriginal and Torres Strait Islander communities, whether sourced indirectly through literature reviews or generated in direct collaboration with communities. Many of the adjustments, methods, and approaches identified in this review are integral to culturally safe and methodologically robust Indigenous health research. However, embedding these approaches within the rigid frameworks and protocols shaped by colonial research systems remains challenging. While the principles of Indigenous health research provide essential guidance, they are not prescriptive, and methods must be responsive to local contexts, cultural protocols, and community priorities. In contrast, reliance on colonial (“Western-centric”) and institutionally governed research models risks marginalizing Indigenous priorities and knowledge systems, leading to epistemological mismatches.

Drawing on over a decade of public health research across New South Wales, Australia, Clapham [154] observes that these approaches often fail to align with Indigenous governance structures, values, and aspirations. These mismatches are compounded by differing modes of knowledge sharing—oral versus written traditions, distinct ways of doing, and diverse knowledge systems [150]. This epistemological disjunction is a global concern. Research frameworks within settler-colonial states routinely exclude Indigenous epistemologies, effectively coercing Indigenous scholars into Western paradigms, undermining epistemic justice and sovereignty [155]. Data practices exemplify this mismatch—colonial data mining often overlooks Indigenous frameworks, giving rise to the data decolonization movement that prioritizes Indigenous paradigms of data governance and self-determination [156]. Although this scoping review actively sought First Nations–specific literature, the processes used to identify, search for, and categorize codesign methods for digital health technologies may not have fully applied an Indigenous lens, highlighting the structural constraints of current research paradigms.

### Implications of Excluding Priority Populations From Digital Health Design

Previous studies have identified structural barriers, such as inadequate infrastructure, poor integration with existing clinical workflows, user-unfriendly health information exchange interfaces, and a lack of trained personnel [157]. At the patient level, barriers include limited broadband access, lack of access to digital tools, and insufficient cultural or linguistic appropriateness of digital health solutions. Additional factors influencing adoption include awareness of digital health solutions, perceived benefits versus burden of technology use, accessibility, trust in digital health technologies, and user experience [158]. These findings highlight how failing to include priority populations in the design of digital health technologies may contribute to lower adoption and reinforce existing health inequities.

### Future Research

Future research should explore the use of existing and new methods focused on the *implementation* and *evaluation* stages of the CeHRes roadmap enabling a more relevant interpretation of the results generated from technology trials. Future research also needs to take into consideration the multifaceted identity of people, developing methods, and adaptions that take into consideration multiple disadvantages and intersectionality. The overlapping social positions and identities held by participants in this research may indicate the need for a specific intersectional theoretical framework to consider how digital health outcomes are influenced by interlocking systems of privilege and oppression [159]. Despite some attempts to validate methods, only 13 (12.3%) studies reported conducting any acceptability review of their methods [36,48,70,74,84,91,97,104,110,111,119,124,129]. Thus, future work should explore using the 5 identified techniques and how we can measure or monitor the impact of equitable approaches in research and development contexts.

### Limitations

There are several strengths and limitations to this review. Key strengths include multiple measures to ensure rigor, such as the involvement of authors from diverse disciplines and from high-, middle-, and low-income countries. The review was guided by a published protocol, incorporated a comprehensive search strategy designed and executed by a specialist librarian (CG), and used dual screening of full-text articles. Data extraction was conducted individually, with verification by a second reviewer (CVR), with disagreements addressed by a third reviewer (KB). Furthermore, the project benefited from oversight by an expert advisory group.

Limitations include the lack of studies from very marginalized priority groups and papers from low- and middle-income countries, and thus we did not analyze papers based on individual priority populations. This was likely because of only English language studies being included. While searching for relevant literature, the information specialist performed searches in 5 mainstream databases, as is common practice. Additional items were identified by citation tracking and searching gray literature sources. However, this search process was faulty because it did not fully compensate for the poor identification and indexing of health literature related to priority populations in the mainstream health databases [155]. As a result, the search did not retrieve 2 relevant articles that had been recommended by one of the authors (AMI) in our review team [122,123]. These 2 items were subsequently included and have improved the review’s coverage for and relevance to specific populations. In the PRISMA flow diagram, the category “References from other sources” could be expanded with an extra dot point: “Items recommended by expert advisors.”

This important lesson prompts us to be mindful of equity issues when conducting literature searches. Moreover, 2 extra resources that may merit wider use in Digital Health Equity research are the proprietary Emerging Sources Citation Index and the open-access website Directory of Open Access Journals. Research should broaden database coverage beyond the dominant biomedical indexing systems and explicitly include Indigenous and regional journals where indigenous research may be more visible. Search strategies should also account for the diverse ways equity concepts are described, moving beyond narrow keyword matches to incorporate culturally grounded terminology. Such steps will reduce the risk of excluding relevant Indigenous-led studies and strengthen the inclusivity and relevance of digital health equity reviews.

### Conclusions

Involving priority communities across the lifecycle of DHT innovation is vital to avoid exacerbating inequities and is possible using the practical methods identified in this research. The most interesting and appropriate methods we identified were developed by priority communities themselves. Thus, we recommend that all digital health projects aiming to provide technology for priority populations include the intended end users in research and development *from the start*, taking on an appropriate knowledge lens (eg, decolonization) and using the adjustments requested by the community to enhance their coproduction experience. An effective way to achieve this is with a community advisor or advisory group. There is also a clear opportunity for educators and certifying organizations to incorporate these approaches into health institutions’ core curricula for digital health competencies addressing structural inequity.

## Supplementary material

10.2196/89596 Multimedia Appendix 1 Search strategies.

10.2196/89596 Multimedia Appendix 2 Charted data.

10.2196/89596 Multimedia Appendix 3 Characteristics of included articles.

10.2196/89596 Multimedia Appendix 4 Overlapping priority population groups.

10.2196/89596 Multimedia Appendix 5 Digital health technology classifications.

10.2196/89596 Multimedia Appendix 6 List of methods to coproduce digital health technology.

10.2196/89596 Multimedia Appendix 7 Recommendations for applying the scoping review findings.

10.2196/89596 Checklist 1 PRISMA-ScR checklist.

## Abbreviations

AI: artificial intelligence
CeHRes: Centre for eHealth Research
DHTs: Digital health technologies
PRISMA: Preferred Reporting Items for Systematic Reviews and Meta-Analyses
PRISMA-ScR: Preferred Reporting Items for Systematic Reviews and Meta-Analyses extension for Scoping Reviews
