# Supplementary material for: Centering Equity During Health Technology Innovation: Scoping Review of Methods and Research Adjustments to Promote Inclusive Coproduction
Source: J Med Internet Res. 2026 Jul 3;28:e89596. doi: 10.2196/89596 (PMC13334495; doi:10.2196/89596)
Supplement: Multimedia Appendix 1 [file jmir-v28-e89596-s001.doc]

# Multimedia Appendix 01 Search Strategies

**Ovid Medline database, 11 Nov 2023:**

1. exp Healthcare Disparities/

2. exp Health Status Disparities/

3. (disparit* or discrimina* or equit* or inequit* or marginali* or underserved).tw,kw,kf.

4. ((cultur* or ethnic* or gender* or racial or relig*) adj3 minorit*).tw,kw,kf.

5. exp *Socioeconomic Factors/

6. (socio-economic adj disadvantage*).tw,kw,kf.

7. exp Digital Divide/

8. Health Literacy/

9. ((digital* adj literate) or (digital adj literac*)).tw,kw,kf.

10. Health Services for Persons with Disabilities/

11. exp *Disabled Persons/

12. ((disabl* or impair*) adj3 (people* or person* or individual* or child* or youth* or population* or worker* or men or women or man or woman or communit* or physical*)).tw,kw,kf.

13. exp Rural Health Services/

14. Rural Health/ or *Rural Population/

15. ((rural or remote) adj3 (population* or communit* or people* or youth* or patient*)).tw,kw,kf.

16. exp Digital Technology/

17. exp Electronic Health Records/

18. mobile applications/

19. telemedicine/ or telehealth/

20. informatics/ or exp consumer health informatics/ or exp dental health informatics/ or exp medical informatics/ or exp nursing informatics/ or exp public health informatics/

21. (digital adj health).tw,kw,kf.

22. (ehealth or e-health or eportal* or e-portal* or mhealth or m-health or (patient adj5 portal*)).tw,kw,kf.

23. (telecare or tele-care or teleconsult* or tele-consult* or telehealth* or tele-health* or telemedicine or tele-medicine).tw,kw,kf.

24. (tool* or framework* or resource* or roadmap* or schema).tw,kw,kf.

25. or/1-15

26. or/16-23

27. 25 and 26

28. 24 and 27

29. (validat* or evaluat*).tw,kw,kf.

30. 28 and 29

31. limit 30 to (English language and yr=”2010-Current”)

**Cochrane Library, 23 Nov 2023**

1. (minorit*):ti,ab,kw

2. MeSH descriptor: [Health Inequities] explode all trees

3. (digital health):ti,ab,kw

4. #1 OR #2

5. #3 AND #4

6. Limit 5 to (English language and yr=”2010-2023”)

**PsycINFO on Ovid database, 2 Dec 2023:**

1. health disparities/

2. (disparit* or discrimina* or equit* or inequit* or marginali* or underserved).ti,ab.

3. ((cultur* or ethnic* or gender* or racial or relig*) adj3 minorit*).ti,ab.

4. exp socioeconomic factors/

5. (socio-economic adj disadvantage*).ti,ab.

6. exp digital divide/

7. exp health literacy/

8. ((digital* adj literate) or (digital adj literac*)).ti,ab.

9. exp Disabilities/

10. ((disable* or impair* or handicap*) adj3 (people* or person* or individual* or child* or youth* or population* or worker* or men or women or man or woman or communit* or physical*)).ti,ab.

11. exp rural health/

12. exp rural environments/

13. ((rural or remote) adj3 (population* or communit* or people* or youth* or patient*)).ti,ab.

14. exp digital technology/

15. exp electronic health records/

16. exp Mobile Applications/

17. exp telemedicine

18. informatics.af.

19. (digital adj health*).ti,ab.

20. (ehealth or e-health or e-portal* or mhealth or m-health or (patient adj5 portal)).mp.

21. (telecare or tele-care or teleconsult* or telehealth* or tele-health* or telemedicine or tele-medicine).ti,ab.

22. (tool or tools or framework* or resource or resources or roadmap* or schema).ti,ab.

23. or/1-13

24. or/14-21

25. 23 and 24

26. 22 and 25

27. (validat* or evaluat*).mp

28. 26 and 27

29. limit 28 to (English language and yr=”2010-Current)

**CINAHL Complete on EBSCOhost, 26 Nov 2023:**

1. MH healthcare disparities

2. MH health status disparities

3. TI (disparit* OR discrimina* OR equit* OR inequit* OR marginali* OR underserved) OR AB (disparit* OR discrimina* OR equit* OR inequit* OR marginali* OR underserved)

4. TI (cultur* OR ethnic* OR gender* OR racial OR relig*) N3 (TI minorit* )) OR AB (cultur* OR ethnic* OR gender* OR racial OR relig*) N3 (TI minorit* ))

5. MM Socioeconomic Factors

6. ((TI socio-economic OR AB socio-economic) W1 (TI disadvantage* OR AB disadvantage*))

7. (MH “Digital Divide+”)

8.(MH “Health Literacy”)

9. (((TI digital* OR AB digital) W1 (TI literate OR AB literate)) OR ((TI digital OR AB digital) W1 (TI literac* OR AB literac*)))

10. (MH “Health Services for Persons with Disabilities”)

11. (MM “Persons with Disabilities+)

12. (((TI disabl* OR AB disabl*) OR (TI impair* OR AB impair*)) N3 ((TI people* OR AB people*) OR (TI person* OR AB person*) OR (TI individual* OR AB individual*) OR (TI child* OR AB child*) OR (TI youth* OR AB youth*) OR (TI population* OR AB population*) OR (TI worker* OR AB worker*) OR (TI men OR AB men) OR (TI women OR AB women) OR (TI communit* OR AB communit*) OR (TI physical* OR AB physical*)))

13. (MH “Rural Health Services+)

14. (MH “Rural Health”) OR (MM “Rural Population”)

15. (((TI rural OR AB rural) OR (TI remote OR AB remote)) N3 ((TI population* OR AB population*) OR (TI communit* OR AB communit*) OR (TI people* OR AB people*) OR (TI youth* OR AB youth*) OR (TI patient* OR AB patient*)))

16. (MH “Digital Technology+”)

17. (MH “Electronic Health Records+”)

18. (MH “mobile applications”)

19. (MH Telemedicine) OR (MH Telehealth)

20. (MH informatics) OR (MH “consumer health informatics+”) OR (MH “dental informatics+”) OR (MH “medical informatics+”) OR (MH “nursing informatics+”) OR (MH “public health informatics+”)

21. ((TI digital OR AB digital) W1 (TI health OR AB health))

22. ((TI ehealth OR AB ehealth) OR (TI e-health OR AB e-health) OR (TI eportal* OR AB eportal*) OR (TI e-portal* OR AB e-portal*) OR (TI mhealth OR AB mhealth) OR (TI m-health OR AB m-health) OR ((TI patient OR AB patient) N5 (TI portal* OR AB portal*)))

23. ((TI telecare OR AB telecare) OR (TI teleconsult* OR AB teleconsult*) OR (TI tele-consult* OR AB tele-consult*) OR (TI telehealth* OR AB telehealth*) OR (TI tele-health* OR AB tele-health*) OR (TI telemedicine OR AB telemedicine) OR (TI tele-medicine OR AB telemedicine))

24. ((TI tool* OR AB tool*) OR (TI framework* OR AB framework*) OR (TI resource* OR AB resource*) OR (TI roadmap* OR AB roadmap*) OR (TI schema OR AB schema))

25. S1 OR S2 OR S3 OR S4 OR S5 OR S6 OR S7 OR S8 OR S9 OR S10 OR S11 OR S12 OR S13 OR S14 OR S15

26. S16 OR S17 OR S18 OR S19 OR S20 OR S21 OR S22 OR S23

27. S25 AND S26

28. S24 AND S27

29. ((TI validat* OR AB validat*) OR (TI evaluat* OR AB evaluat*))

30. S28 AND S29

31. S28 AND S29, limiters publication date 2010-20231231

32. S31, limited to English language.

**Web of Science Core Collection search, 2 Feb 2023:**

1. “digital health equit* (Title)

2. Limit #1 to Review Article.

[END of Search details]
