# Supplementary material for: Centering Equity During Health Technology Innovation: Scoping Review of Methods and Research Adjustments to Promote Inclusive Coproduction
Source: J Med Internet Res. 2026 Jul 3;28:e89596. doi: 10.2196/89596 (PMC13334495; doi:10.2196/89596)
Supplement: Multimedia Appendix 3 [file jmir-v28-e89596-s003.doc]

# Multimedia Appendix 03 Characteristics of Included Articles

| **First Author** | **Year** | **Country where study was based** | **Country income level** | **Study Design** | **Aim** | **Funding body** |
| --- | --- | --- | --- | --- | --- | --- |
| Aladin et al. | 2023 | United States | High income | Demonstration Project | The study aims to develop and describe the early implementation of a digital intervention combining a mobile app, peer navigation, and a digital comic to improve HIV care outcomes for young adults. | Funded by award number H97HA28890 from the U. S. Department of Health and Human Services, Health Resources and Services Administration (HRSA), HIV/AIDS Bureau’s Special Projects of National Significance Program (SPNS). |
| Albright et al. | 2015 | United States | High income | Qualitative | The study aims to design and evaluate a text messaging program with the goal of increasing participant engagement and retention in the Colorado Healthy Heart Solutions (CHHS) program. | Funded by the Agency for Healthcare Research & Quality, #1 P01 HS021138, “Improving Cardiovascular Screening and Management Through a Bidirectional Personal and Technological Interface.” |
| Almond et al. | 2016 | Australia | High income | Qualitative | The study aims to report the findings from people with complex chronic conditions experiences of MyHR that may contribute to improving the adoption, use and utility of Australia’s implementation and evaluation of MyHR. | Not provided. |
| Almond et al. | 2017 | Australia | High income | Qualitative | The study aims to investigate the adoption, use, and utility of MyHR in rural remote Australia community settings and develop an approach for improving national roll out of MyHR. | Not provided. |
| Antonelli et al. | 2021 | United States | High income | Qualitative | The study aims to develop the 4to24 app, which provides information, resources, and activities to help parents support their children with visual impairments (aged 4-24) in achieving independence and employment as adults. | Fundy by the U.S. Department of Health and Human Services, National Institute on Disability, Independent Living, and Rehabilitation Research (NIDILRR) under grant 90RT5040. |
| Aronoff-Spencer et al. | 2022 | United States | High income | Qualitative | The study aims to design and implement a participatory framework to produce effective health care solutions through co-design with diverse stakeholders. | Funded by the ICF Macro, Inc, to conduct LAUNCH (Linking and Amplifying User-Centered Networks through Connected Health)–related activities (2018-2019). Amgen provided funding for the University of California San Diego contributions, whereas the Federal Communications Commission provided funding for the Connect2HealthFCC Task Force activities. |
| Baik et al. | 2023 | United States | High income | Qualitative | The study aims to design and implement two culturally, informed, bilingual, evidence-based psychosocial smartphone application called My Guide and My Health designed to improve the health-related quality of life (HRQOL) of American Hispanic/Latina women who are survivors of breast cancer (BCS). | Not provided. |
| Bauer et al. | 2018 | United States | High income | Qualitative | The study aims to incorporate a mHealth platform to deliver integrated primary care to rural patients with posttraumatic stress disorder and/or bipolar disorder. | Funded by the Patient-Centered Outcomes Research Institute (PCORI) Award, The John A. Hartford Foundation, National Institutes of Health. |
| Bendixen et al. | 2017 | United States | High income | Qualitative | The study aims to gather data from persons with brain and spinal cord anomalies (BSA) and their caregivers to better understand how mHealth would be most helpful in supporting them to proactively manage daily self-care routines and to access medical care as needed. | Funded by grants from the National Institute on Disability, Independent Living, and Rehabilitation Research (NIDILRR; grant numbers 90DP0064 and 90RE5018), which is a center within the Administration for Community Living (ACL), US Department of Health and Human Services (HHS). |
| Blackwell et al. | 2020 | United States | High income | Mixed Methods | The study aims to explore urban African American and Afro-Caribbean immigrant pregnant women's experiences with prenatal care, mHealth usage, and changes in knowledge, perceptions, and behavior after the T4B mHealth intervention. | Funded by SUNY Downstate Medical Center President’s Health Disparities fund. |
| Bounds et al. | 2023 | United States | High income | Case Study | The study aims to adapt the four-box model to guide mHealth developers though ethical considerations when designing mHealth interventions for teens with adverse childhood experiences. | Not provided. |
| Bravo et al. | 2014 | United States | High income | Mixed Methods | The study aims to determine the acceptability and usability of a novel mHealth tool through semi-structured interviews, survey, and participant observation at a public hospital breast-imaging center serving a diverse, low-income population. | Funded by the University of California at San Francisco Center for Aging in Diverse Communities grant no. P30-AG15272 under the Resource Centers for Minority Aging Research program of the National Institute on Aging. |
| Brewer et al. | 2019 | United States | High income | Mixed Methods | The study aims to describe the iterative process of design and development of the FAITH! App prototype through a mixed-methods CBPR approach and to provide the initial results of a pilot study assessing its acceptability, usability, and user satisfaction. | Funded by the Building Interdisciplinary Research Careers in Women’s Health Scholars Program (award number K12HD065987-07) from the National Institutes of Health (NIH) Office of Research on Women’s Health, Mayo Clinic Women’s Health Research Center, and the National Center for Advancing Translational Sciences (Clinical and Translational Science Awards Grant Number KL2 TR002379), a component of the NIH. This study was further supported by the Mayo Clinic Center for Innovation, Mayo Clinic Center for Translational Science Activities (UL1TR000135), Mayo Clinic Department of Cardiovascular Medicine, and Mayo Clinic Office of Health Disparities Research. |
| Brooks et al. | 2021 | Indonesia | Upper middle income | Qualitative | The study aims to report on the development of the IMPeTUS intervention designed to improve mental health literacy amongst children and young people aged 11–15 in Java, Indonesia. | Funded by the MRC/DFID/NIHR/ESRC programme of research to improve adolescent health in low- and middle-income countries. (MR/ R022151/1). |
| Buckingham et al. | 2023 | United Kingdom | High income | Mixed Methods | The study aims to produce an information and training resource that could be used by practitioners and patients, to improve knowledge, skills, and confidence in telephone and video-based consultations for physical disabilities and movement impairment. | Funded by UK Research and Innovation-Medical Research Council. (UKRI-MRC; award COV0079). |
| Burchert et al. | 2018 | Germany  Sweden  Egypt | High income  High income  Lower middle income | Qualitative | The study aims to understand and evaluate access, usage, and potential barriers regarding an e-mental health intervention (Step-by-Step). | Funded by European Union’s Horizon 2020 Research and Innovation Program Societal Challenges under grant agreement No 733337. |
| Calderon et al. | 2017 | Peru | Upper middle income | Qualitative | The study aims to contribute a qualitative formative evaluation of a proposed mHealth program designed to support caregivers of young children in a resource-limited setting. | Funded by the Penn-Peru Pilot Grant Program of the University of Pennsylvania and the Universidad Peruana Cayetano Heredia. |
| Campbell et al. | 2017 | Uganda | Low income | Qualitative | The study aims to propose a novel conceptual framework for technology acceptance for mHealth applications targeted to low-literacy populations in resource low settings. | Funded by the National Institutes of Health (R24 TW007988, T32 AI007433, K23 MH099916), and the Harvard Global Health Institute. |
| Carolan-Olah et al. | 2021 | Australia | High income | Mixed Methods | The study aims to develop and evaluate an eHealth intervention to promote healthy lifestyle for pregnant women. | Funded by the Lord Mayor’s Charitable Foundation. |
| Castillo et al. | 2022 | United States | High income | Qualitative | The study aims to adapt and translate an English-language pregnancy mobile app to meet the cultural and linguistic needs of Spanish-speaking Latino immigrants. | Funded by the Centers for Disease Control and Prevention (Grant Number: R44DP006417) and the National Institutes of Health (Grant Number: KL2 TR001856). |
| Ceasar et al. | 2019 | United States | High income | Qualitative | The study aims to utilize focus groups for gathering qualitative data to inform the development of an app that promotes physical activity among African American women in Washington, DC. | Funded by the National Heart, Lung, and Blood Institute, National Institute on Minority Health and Health Disparities, NIH Medical Research Scholars Program, Doris Duke Charitable Foundation, American Association for Dental Research, Colgate-Palmolive Company, Genentech, Elsevier, and other private donors. |
| Cerda Diez et al. | 2019 | United States | High income | Mixed Methods | The study aims to adapt and evaluate a digital family health history tool, VICKY (VIrtual Counselor for Knowing Your Family History), for Spanish speakers. | Funded by the National Human Genome Research Institute. |
| Champoux et al. | 2020 | United States | High income | Qualitative | The study aims to describe the process used to design and refine text messages for use in the Reach Out mobile intervention, which aimed to reduce blood pressure in primarily African American hypertensive patients who were evaluated in a safety net emergency department in Flint, Michigan. | Funded by a grant from the National Institutes of Health/National Institute on Minority Health and Health Disparities (R01MD011516). |
| Chandler et al. | 2023 | United States | High income | Mixed Methods | The study aims to evaluate usability, acceptability, and engagement of the Savvy HER (Sexual/HIV Health Electronic Empowerment Resource) app. | Funded by the National Institutes of Mental Health Grant number 5R34MH128048-02. |
| Chee et al. | 2017 | United States | High income | Mixed Methods | The study aims to determine the preliminary efficacy of a culturally tailored registered nurse (RN) moderated Internet Cancer Support Group for Asian American breast cancer survivors (ICSG-AA) in enhancing the women’s breast cancer survivorship experience. | Funded by the Population Science Pilot Project Award, the NCI Cancer Center Support Grant (P30 CA016520) and the Abramson Cancer Center of the University of Pennsylvania. The Chinese translation process  involved in the study was also funded by the Chang Gung Medical Research Foundation (BMRPA50 &  ZZRPF3C0011). |
| Cheng et al. | 2020 | Australia | High income | Mixed Methods | The study aims to determine if the Ophelia process can be adapted into the digital context and applied to co-design solutions addressing eHealth literacy needs. | Funded by the Deakin University Higher Degree Research Grant; Deakin University Postgraduate Research Scholarship; National Health and Medical Research Council (NHMRC) of Australia Principal Research Fellowship. |
| Dal Bello-Haas et al. | 2014 | Canada | High income | Mixed Methods | The study aims to examine the demand, acceptability, practicality, and implementation of telehealth-delivered exercise for rural, community-dwelling individuals diagnosed with dementia and their caregivers. | Funded by the Ralston Brothers Medical Research Fund, College of Medicine, University of Saskatchewan. |
| Dang et al. | 2023 | Australia | High income | Qualitative | The study aims to detail the co-design approach and report the views of workshop participants regarding the cultural adaptation of iSupport Lite for a multicultural Australian setting. As well as to assess the acceptability and intention to use in terms of performance expectancy, effort expectancy, social influence, and facilitating conditions. | Funded by the Medical Research Future Fund (MRFF), Australian Government Department of Health (grant number APP2008065). |
| Day et al. | 2021 | United States | High income | Mixed Methods | The study aims to describe the Personalized Implementation of Video Telehealth for Rural Veterans (PIVOT-R) approach, developed in response to the unique needs of rural veterans. | Funded by the VA Office of Rural Health, Veterans Rural Health Resource Center-Salt Lake City and partly supported by the use of facilities and resources of the Houston VA HSR&D Center for Innovations in Quality, Effectiveness and Safety (grant number CIN13-413) and the VA South Central Mental Illness Research, Education and Clinical Center. |
| Day et al. | 2023 | United States | High income | Mixed Methods | The study aims to describe the Personalized Implementation of Video Telehealth for Rural Native Veterans approach and examine the utility of implementation science for dissemination of the culturally centered model of mental health care for rural native veterans. | Funded by grants from the VA Office of Rural Health and Veterans Health Resource Center-Salt Lake City. |
| Dobson et al. | 2017 | New Zealand | High income | Mixed Methods | The study aims to assess the engagement of participants with the TextMATCH program, including enrollement and disengagement rates, as well as, assess the acceptability of the program including its cultural appropristeness and relevance, reasons for withdrawal, and to determine ways in which the program could be improved | Funded by the New Zealand Ministry of Health via contract with Waitemata and Auckland District Health Boards. |
| Doty et al. | 2020 | United States | High income | Qualitative | The study aims to explore Latinx parents’ access to technology, current use of technology, and intentions to use a parenting app, based on a mixed methods contextual inquiry; as well as to identify the stakeholder values and parent desires for a parenting/stress reduction app and wearable device to support awareness of stress levels that could affect parenting; and to synthesize and integrate parent feedback about the mobile app mockup and the working prototype into the design of the mobile app. | Funded by the Family Process Institute and the Institute for Translational Research at the University of Minnesota. JD was supported by the Health Resources and Services Administration of the US Department of Health and Human Services under National Research Service Award in Primary Medical Care grant number T32HP22239 (Principle Investigator IB, Bureau of Health Workforce). |
| Enyioha et al. | 2023 | United States | High income | Qualitative | The study aims to investigate the preferences of Black smokers for features of mHealth apps for smoking cessation using QuitGuide as a reference. | Funded by the National Center for Advancing Translational Sciences (NCATS), National Institutes of Health (NIH), through grant award number UL1TR002489. |
| Fontil et al. | 2016 | United States | High income | Qualitative | The study aims to adapt a digital health program to prevent diabetes among low-income patients through a real-world, user-centered process and test its feasibility in prediabetic patients at a large, urban, county-operated safety net clinic. | Funded by Omada Health, Inc. a company that makes and owns online behavior change programs. |
| Garvelink et al. | 2020 | Canada  France | High income  High income | Qualitative | The study aims to perform an in-depth exploration among potential end users about how to improve the interactive website to better inform older adults and caregivers about ways to stay independent at home. | Funded by Improving Cognitive and Joint Health Network of the Canadian Institute of Health Research. |
| Givoenco et al. | 2021 | United States | High income | Qualitative | The study aims to create a toolbox to provide mHealth interventions for youth with or in risk of HIV infection. | Funded by Eunice Kennedy Shriver National Institute of Child Health and Human Development of the National Institutes of Health and the National Institute of Mental Health of the National Institutes of Health. |
| Godleski et al. | 2020 | United States | High income | Qualitative | The study aims to determine the health information needs of the target population and evaluate the feasibility of information shared through an app. | Funded by a Rochester’s Child - Rochester Area Community Foundation Grant (20183010). |
| Gordon et al. | 2016 | United States | High income | Qualitative | The study aims to describe an iterative participatory design strategy that resulted in a suite of 3 eHealth tools geared to the support needs of low-income, ethnic/racial minority women at risk for perinatal mental disorders. | Funded by the Agency for Healthcare Research and Quality grant No. 1 K18 HS022441-01 and the Penn Medicine Center for Innovations. |
| Greenhalgh et al. | 2015 | United Kingdom | High income | Qualitative | The study aims to define quality in telehealth and telecare to enhance the well-being of patients using assistive technologies while living with illness or disabilities. | Funded by Technology Strategic Board under its Assisted Living Innovation Platform (2797-25242/400217). NIHR Senior Investigator Award. |
| Grewal et al. | 2023 | United States | High income | Demonstration Project | The study aims to describe major activities involved with developing, implementing, and evaluating a telemedicine program specifically designed to provide health- care services to persons with HIV. | Funded by National Center for HIV/AIDS, Viral Hepatitis, STD, and TB Prevention. |
| Ha et al. | 2023 | Korea, Rep. | High income | Qualitative | The study aims to conceptualize, develop, and validate a digitally integrated health care service platform for people with disability, caregivers, and health care professionals, using Internet of Things devices and patient generated health data to contribute to improving digital health equity. | Funded by Rehabilitation Research & Development Support Program (NRCRSP-EX21005) of the National Rehabilitation Center, Ministry of Health and Welfare, Korea. |
| Handley et al. | 2016 | United States | High income | Qualitative | The study aims to tailor an IT-enabled health communication program to promote DPP-concordant behavior change among postpartum Latina women with recent gestational diabetes. | Funded at least in part with Federal funds from the U.S. Department of Agriculture. |
| Harris et al. | 2023 | United States | High income | Qualitative | The study aims to explore COVID-19 information behavior among Black Americans, including their preferences for education via mobile health (mHealth), barriers and facilitators to education and testing, and key content for a COVID-19 mHealth app. The study was the initial phase of a larger project to develop, pilot, and evaluate a mobile health intervention for a population at high risk for COVID-19 and cardiovascular comorbidities. | Funded by the Association of Black Cardiologists Innovation Award and the National Institutes of Health (NIH) Clinical and Translational Science Award (CTSA) program. |
| Hearn et al. | 2022 | Uganda | Low income | Mixed Methods | The study aims to develop a digital health intervention that enables improved self-care amongst heart failure patients in Uganda. | Funded by the Ted Rogers Centre for Heart Research, Wolfond Chair in Digital Health, Fogarty International Center. |
| Henson et al. | 2023 | Australia | High income | Qualitative | The study aims to identify how older Aboriginal and Torres Strait Islander women use digital health technologies to enhance health, illuminate case studies of how older women have used digital technologies to enhance health, and develop a working model to inform the development and implementation of digital health technologies that are acceptable and useful for older Aboriginal and Torres Strait Islander women. | Funded by Macquarie University (20213896; allocation for Doctor of Philosophy students). |
| Higa et al. | 2021 | United States | High income | Qualitative | The study aims to pilot a program integrating family and friend support, community health services, telehealth-enabled diabetes self-management (DSM) education, and mobile technologies in an under-resourced, rural community in Hawai’i. | Funded by University of Hawai’i faculty research funds. |
| Hoque et al. | 2017 | Bangladesh | Lower middle income | Quantitative | The study aims to develop a theoretical model based on the Unified Theory of Acceptance and Use of Technology (UTAUT) and then empirically test it for determining the key factors influencing elderly users’ intention to adopt and use the mHealth services. | Funded by seed research grant of School of Business and Tourism (SBaT), Southern Cross University, under grant No. (31496). |
| Howells et al. | 2022 | United Kingdom | High income | Qualitative | The study aims to explore the experience and impact of organisational and technology changes in response to COVID-19 on access to health care for people experiencing homelessness. | Funded by National Institute for Health Research (NIHR) through the Greater Manchester Patient Safety Translational Research Centre. |
| Hughes et al. | 2018 | United States | High income | Mixed Methods | The study aims to identify design and functionality issues, along with usability problems, of a prototype of the outREACH tele-rehabilitation app designed for underserved population. | Funded by the San Francisco State University (SFSU) Center for Computing Life Sciences (CCLS) Mini Grant. |
| Hutchings et al. | 2022 | United Kingdom | High income | Mixed Methods | The study aims to test digital innovations in a real-world setting to understand the factors that support patient and staff engagement in the use of digital health care innovations. | Funded by Nuffield Trust an independent health think tank. |
| Hynie et al. | 2022 | Canada | High income | Qualitative | The study aims to examine the multidimensional nature of access to virtual mental health (VMH) care for refugee newcomers during the COVID-19 pandemic, using Levesque et al.’s Client-Centered Framework for Assessing Access to Health Care. | Funded by a grant from the Canadian Institutes of Health Research (#173101) in partnership with the Ontario Ministry of Health and Long Term Care (#714). |
| Jenness et al. | 2022 | United States | High income | Case Study | The study aims to develop technology-supported, evidence-based psychosocial tools to improve access to, engagement in, and scalability of mental health care for teens. | Funded by the University of Washington ALACRITY Center under National Institute of Mental Health Award #1P50MH115837, and supported by the National Center for Advancing Translational Sciences of the National Institutes of Health under Award Number UL1 TR002319 and the National Institute of Mental Health (K23MH112872). |
| Jiam et al. | 2017 | United States | High income | Mixed Methods | The study aims to develop and evaluate the important information about me (IIAE) app, which supports adults, children, and families with neurodevelopmental disabilities by allowing them to manage and communicate healthcare information. | Funded by the Kennedy Krieger Institute. |
| Kang et al. | 2023 | Taiwan, China | High income | Qualitative | The study aims to describe the development of a mobile application of the PmP Traditional Chinese version (PmP-C) and evaluated its usability. | Funded by the Ministry of Sciences and Technology, Taiwan under (Grants MOST 107-2314-B-182048 and 108-2314-B-182 041-MY2). |
| Kayastha et al. | 2021 | Nepal | Lower middle income | Qualitative | The study aims to understand the attitudes of women about mobile games, understand the acceptability and usability of the prototype MANTRA intervention by women and FCHVs and to explore their perceptions of knowledge change brought about by the mHealth gaming application. | Funded by the United Kingdom Research Councils Grand Challenges Research Fund [Project 538621 Award: 173142] NERC Reference: NE/P016103/1. |
| Kothari et al. | 2020 | United States | High income | Qualitative | The study aims to understand the health information needs of low-income mothers and their perceptions about using a parenting app to access parenting and nutrition information. | Funded by the Rochester’s Child – Rochester Area Community Foundation Grant. |
| Lindegaard et al. | 2022 | Sweden | High income | Mixed Methods | The study aims to understand if internet based cognitive therapy (ICBT) is a feasible and acceptable treatment for adolescents and young adults with a refugee background. | Funded by a grant from the Swedish Research Council, “Development and evaluation of internet-supported treatment for recently arrived children and adolescents with psychiatric problems.” |
| Liu et al. | 2019 | United States | High income | Mixed Methods | The study aims to describe the development and usability evaluation of the "My Wheelchair Guide" app. | Funded by the National Institute of Disability, Independent Living, and Rehabilitation Research. |
| Luo et al. | 2021 | United States | High income | Qualitative | The study aims to explore the potential use of smartphone apps for diabetes self-management in an underserved population, focusing on user willingness and identifying barriers and facilitators to app adoption. | This research received no external funding. |
| Mafalda et al. | 2020 | Portugal | High income | Mixed Methods | The study aims to design and build instructional content on feeding patients in home care for a digital platform that supports family caregivers, in a context of low health literacy. | Not provided. |
| Maragh-Bass et al. | 2022 | United States | High income | Mixed Methods | The study aims to empower young Black adults in COVID-19 vaccine decision-making through the creation of digital storytelling. | Funded by the National Institute for Minority Health and Health Disparities. |
| Mauka et al. | 2021 | Tanzania | Lower middle income | Qualitative | The study aims to present the development process of a mobile health (mHealth) application to improve adherence to pre-exposure prophylaxis (PrEP) against HIV among men who have sex with men (MSM) and female sex workers (FSW) in Tanzania. | Funded by the Research Council of Norway through the global health and vaccination (GLOBVAC) program and the European and Developing Countries Clinical Trials Partnership (EDCTP2) program supported by the European Union. |
| Mayberry et al. | 2016 | United States | High income | Qualitative | The study aims to develop a family-focused intervention acceptable to patients receiving care from Federally Qualified Health Centers (FQHCs), obtain feedback and data to improve the intervention, and ensure our research processes were sound prior to an evaluative trial. | Funded by the National Institute of Diabetes and Digestive and Kidney Diseases (NIDDK) through Dr. Mayberry’s Pilot and Feasibility Award from the Vanderbilt Center for Diabetes Translational Research. |
| McCall et al. | 2021 | United States | High income | Mixed Methods | The study aims to evaluate the usability of the prototype of an app that is designed for supporting the self-management of anxiety and depression in African American women. | The first author (TM) was supported by funding from the National Library of Medicine’s Institutional Training Grant for Research Training in Biomedical Informatics and Data Science at the Carolina Health Informatics Program (T15LM012500) and Yale Center for Medical Informatics (T15LM007056). This research was also supported by the Intramural Research Program of the National Library of Medicine, National Institutes of Health. |
| McCall et al. | 2022 | United States | High income | Qualitative | This study aims to assess what types of content, features, and important considerations should be included in the design of a mobile app tailored to support management of anxiety and depression among Black women. | TM and MP were supported by funding from the National Library of Medicine (NLM) under award number T15LM012500 during the time the study was conducted. TM is currently supported by funding from the NLM under award number R01LM013477. MT is supported by the National Institute of Mental Health of the National Institutes of Health under award number R25MH087217. |
| Meijer et al. | 2021 | Netherlands | High income | Mixed Methods | The study aims to evaluate StopCoach, a mobile phone delivered eHealth intervention (app) targeted at lower-SES smokers based on StopAdvisor, in a real-world setting. We aimed to implement StopCoach in blended care settings within five municipalities in The Netherlands. | Funded by a Grant provided by the Noaber Foundation. |
| Merculieff et al. | 2021 | United States | High income | Mixed Methods | The study aims to describe a two-phased approach to develop content for Connecting Alaska Native People to Quit Smoking, a Facebook group intervention to reduce barriers to evidence-based smoking cessation treatment for AN people in Alaska. | Funded by the National Institute on Drug Abuse (NIDA) of the National Institutes of Health under Award Number R34DA046008 (Patten). |
| Miah et al. | 2017 | Bangladesh | Lower middle income | Mixed Methods | The study aims to design and evaluate an innovative mobile decision support system (MDSS) solution for rural citizens healthcare decision support and information dissemination. | Not provided. |
| Morrow et al. | 2017 | United States | High income | Mixed Methods | The study aims to design and evaluate EMR portal messages that support patient-centered self-care, especially for older adults with diverse literacy and numeracy abilities. | Funded by the Agency for Healthcare Research and Quality (grant number R21HS022948). |
| Mueller et al. | 2020 | Nepal | Lower middle income | Mixed Methods | The study aims to develop, pilot, and assess a serious game for mobile devices that teaches geohazard, maternal, and neonatal health messages. | Funded by the United Kingdom Research Councils Global Challenges Research Fund (Project: 538621 Award: 173142). |
| Nouri et al. | 2019 | United States | High income | Qualitative | The study aims to describe variations in patients’ engagement in the app design process, focusing on limited health literacy (LHL), limited English proficiency (LEP), and limited digital literacy (LDL). | Funded by the Agency for Healthcare Research and Quality (1R01HS025429-01), a career development award by the National Institute of Mental Health (5K23MH0944442), a career development award by the National Cancer Institute (K24CA212294), and a research training grant by the National Research Service Award (T32HP19025). |
| Ospina-Pinillos et al. | 2019 | Australia | High income | Qualitative | The study aims to to (1) conduct co-design workshops with end users to co-design and culturally adapt the mental health e-clinic (MHeC) for Spanish-speaking young people based in Australia; (2) inform the development of the alpha prototype of the MHeC-S; (3) test the usability of the alpha prototype of the MHeC-S; (4) translate, culturally adapt, and face-validate the self-report assessment to a Spanish-speaking population based in Australia; and (5) collect information to inform the beta prototype of the MHeC-S. | Funded by the Young and Well Cooperative Research Centre (Western Sydney University, Penrith, Australia; 2014-2016). |
| Owens et al. | 2020 | United States | High income | Qualitative | This study aims to qualitatively assess the cultural sensitivity of the Breathe Easier mobile application. | Funded by the Bristol Myers Squibb Foundation. |
| Pathak et al. | 2021 | United States | High income | Mixed Methods | The study aims to develop motivational text messages in English and Spanish for a smartphone app-based intervention to encourage physical activity in low-income minority patients with diabetes and depression. | Not provided. |
| Peng et al. | 2022 | Malaysia | Upper middle income | Qualitative | The study aims to identify barriers and facilitators of AI chatbot acceptance for HIV prevention among Malaysian men who have sex with men (MSM). | Funded by research grants from the National Institutes of Health (R21 TW011663 for ZN and FLA; R21 AI152927 for JAW, FLA, and ZN; and R21 TW011665 for RS) and the Fund for Lesbian and Gay Studies (FLAGS) Research Award at Yale University. |
| Petros De Guex et al. | 2023 | United States | High income | Mixed Methods | The study aims to determine how mHealth intervention can address the challenges that Latinx people with HIV (PWH) in the United States face. | Funded by Virginia Department of Health through Ryan White Part B special projects funding. |
| Pipicella et al. | 2023 | Australia  New Zealand | High income  High income | Qualitative | The study aims to develop and implement a consensus method between clinicians, parents, and children with inflammatory bowel disease to vote on pediatric metrics to be included in the Crohn’s Colitis Care eHealth platform prior to software build. The objective was to ensure consumer and clinician needs were met. | Funded by the Leona M. and Harry B. Helmsley Charitable Trust (Grant No. #2002- 04267) and The Australian Government, Department of Education and Training: Research Training Program (RTP) Fees Offset Scholarship. |
| Pluye et al. | 2020 | Canada | High income | Mixed Methods | The study aims to (1) compare the low-SES mothers’ perception of outcomes of web-based parenting information with that of other mothers, and (2) explore the perspective of low-SES mothers on contextual factors and information needs and behavior associated with these outcomes. | Funded by the Canadian Institutes of Health Research (CIHR #201610PJT-377359-PJT-CFAA-109294) and N&G (Foundation Lucie & André Chagnon). |
| Povey et al. | 2020 | Australia | High income | Mixed Methods | The study aims to explore the lived experience of mental health and wellbeing with Aboriginal and Torres Strait Islander youth in three Top End Northern Territory (NT) settings, examine the characteristics of e-mental health resources that render them acceptable and appropriate for Aboriginal and Torres Strait Islander youth, and draft a culturally responsive e-mental health resource in collaboration with Aboriginal and Torres Strait Islander youth participants. | First author supported by Australian Government Research Training Program (RTP) Scholarship and an Australian Rotary Health, Ian Scott Scholarship. |
| Radcliffe et al. | 2021 | United States | High income | Mixed Methods | The study aims to develop and test a prototype app accessibility testing protocol and to disseminate findings via an app curation website that provides information to people with disabilities about mHealth app suitability, based on their needs, impairments, and mobile device access methods. | Funded by the National Institute on Disability, Independent Living and Rehabilitation Research (NIDILRR) of the U.S. Department of Health and Human Services, Grant number 90DPHF004. |
| Resnick et al. | 2022 | United States | High income | Mixed Methods | The study aims to assess the usability and acceptability of a cancer prevention and goal setting app among non-Hispanic Black patients. | Funded by American Cancer Society and Abramson Cancer Center (ACC) grants. One author supported by the American Cancer Society—Tri-State CEO’s Against Cancer Mentored Research Scholar Grant and the 2018 ACC Population Science Pilot Award Program, ACC’s Cancer Center Support Grant. |
| Robbins et al. | 2019 | United States | High income | Mixed Methods | The study aims to develop a tailored website to promote awareness about obstructive sleep apnea (OSA) among community-dwelling black/African-American individuals using mixed methods including in-depth interviews, usability-testing procedures, and brief surveys. | Funded by the National Heart, Lung, and Blood Institute (NHLBI). |
| Rozbroj et al. | 2015 | Australia | High income | Qualitative | The study aims to determine how e-therapies for depression and anxiety could be improved to address the therapeutic needs of lesbians and gay men. | Funded by a grant awarded as part of the “National Priority Driven Research Program in Gay, Lesbian, Bisexual, Transgender, and Intersex (GLBTI) People.” |
| Russ et al. | 2021 | United Kingdom | High income | Qualitative | The study aims to evaluate acceptability of the MySurgery app; barriers and facilitators to accessing and using the app; ideas to aid implementation, and to analyze how app use might differ for diverse patients. | Funded by the National Institute for Health Research (NIHR) Applied Research Collaboration South London (ARC South London) at the King’s College Hospital NHS Foundation Trust; the NIHR through a Knowledge Mobilization Fellowship; an Improvement Science Fellowship at the Health Foundation; King’s Health Partners (Guy’s and St Thomas’NHS Foundation Trust, King’s College Hospital NHS Foundation Trust, King’s College London, and South London and Maudsley NHS Foundation Trust), Guy’s and St Thomas’ Charity, the Maudsley Charity, and the Health Foundation. |
| Shrestha et al. | 2023 | Malaysia | Upper middle income | Mixed Methods | The study aims to assess the usability of JomPREP and acceptability among MSM living in the Greater Kuala Lumpur region, Malaysia. | Funded by a career development and research award from the National Institute on Drug Abuse (K01 DA051346) and a research award from the Fogarty International Center (R21TW011665) for Roman Shrestha. |
| Simons et al. | 2018 | Belgium | High income | Mixed Methods | The study aims to describe the development, usability, acceptability, and feasibility of a new theory- and evidence-based smartphone app to promote an active lifestyle in lower-educated working young adults. | Author DS supported by a PhD fellowship of The Research Foundation-Flanders (11U8114N). Author KDC supported by the Research Foundation Flanders (FWO) (postdoctoral research fellowship: FWO11/PDO/097). Author CV supported by a National Heart Foundation of Australia Future Leader Fellowship (ID 100427). |
| Spanhel et al. | 2019 | Germany | High income | Qualitative | The study aims to investigate user experience to identify elements of Internet-based interventions for sleeping problems that need cultural adaptation to be suitable for refugees. | No specific grant from funding agencies in the public, commercial, or not-for-profit sectors. |
| Sun et al. | 2020 | United States | High income | Mixed Methods | The study aims to develop and test the usability and acceptability of a prototype mobile app to promote HIV prevention among transgender women. | Funded by grant R43MD012279 from the National Institute on Minority Health and Health Disparities and K12HS022981 from the Agency for Healthcare Research and Quality. |
| Swallow et al. | 2016 | United Kingdom | High income | Mixed Methods | The study aims to (1) identify gaps in current online, chronic kidney disease-specific information and support, and determine the desirable components of the online parent information and support (OPIS) application, (2) develop the OPIS to address these identified needs and (3) implement OPIS and assess feasibility and methods in a small-scale randomized controlled trial (RCT) of OPIS. | This article presents independent research commissioned by the National Institute for Health Research (NIHR) under the Research for Patient=44 V. Swallow et al. benefit programme (PB-PG-0110-21305). |
| Tonkin et al. | 2017 | Australia | High income | Mixed Methods | The study aims to consult remote Indigenous community (RIC) members to inform the development of a smartphone app that can be used to monitor and reduce sugar sweetened beverage (SSB) intake in RICs. | Funded by the National Health and Medical Research Council project grant (631947); project titled “Improving Chronic Disease Outcomes for Indigenous Australians: Causes, Interventions, System Change.” JB was supported by a National Heart Foundation Future Leader Fellowship (100085). TW was supported by a National Health and Medical Research Council Early Career Fellowship (1053359). CM was supported by a National Heart Foundation Postdoctoral Fellowship (100188). BC was supported by the Northern Territory Government Department of Health under the Indigenous Cadetship program. |
| Tremblay et al. | 2021 | Canada | High income | Qualitative | The study aims to assess the usability and user experience of a co-designed eHealth prototype to support caregivers of functionally dependent older persons in their help-seeking process. | Funded by the Québec amis des aînés program of the Ministère de la Famille (Québec). |
| van den Bergh et al. | 2023 | Netherlands | High income | Mixed Methods | The study aims to describe the experiences of people with Parkinson's disease and physiotherapists who used a remote monitoring system in daily clinical practice. | Funded by the Center of Excellence grant from the Parkinson’s Foundation. RB and MM were supported by the Gatsby Foundation [GAT3676] as well as by the Ministry of Economic Affairs by means of the PPP Allowance made available by the Top Sector Life Sciences & Health to stimulate public- private partnerships. This activity has received funding from the European Institute of Innovation and Technology (EIT). This body of the European Union receives support from the European Union’s Horizon 2020 research and innovation program. The Vital@Home project was part of EIT Digital, grant numbers 17158 and 18146. |
| Van Dooren et al. | 2023 | Belgium | High income | Qualitative | The study aims to develop and evaluate an mHealth app to bridge the communication gap between people with 22q11 deletion syndrome (22q11 DS) and their caregivers. The study also sought to identify key design principles for developing apps for people with cognitive impairments, and to provide insights into how to maximize the potential of digital tools for this population. | Not provided. |
| Vangeepuram et al. | 2018 | United States | High income | Quantitative | The study aims to evaluate the usage of and attitudes towards mobile technologies of individuals from low-income and racial/ethnic minority backgrounds to inform the development of a new mobile platform for dissemination of diabetes prevention information. | Funded by the National Institute on Minority Health and Health Disparities (Grant number 5R24MD001691-11) |
| Velez et al. | 2014 | Ghana | Lower middle income | Mixed Methods | The study aims to evaluate a clinical prototype (of mClinic a mobile health application for midwives) via usability testing to identify development flaws and required software enhancements. | Funded by the National Institute of Nursing Research (P30NR010677), Health Services Resource Administration (1D11 HP07346), International Development Research Centre, Rockefeller Foundation, Novartis Fund for Sustainable Development, OpenROSA Consortium, Jonas Center for Nursing Excellence, and the National Library of Medicine (5 T15 LM007079-20). |
| Verbiest et al. | 2019 | New Zealand | High income | Qualitative | This study aims to describe the codesign methods and processes used in  the OL@-OR@ project and describe how codesign was used to inform and build a theory-driven approach to the selection of behavioral determinants and change techniques as part of the intervention. | Funded by the Healthier Lives He Oranga Hauora National Science Challenge. |
| Wagner et al. | 2023a | United States | High income | Qualitative | This work describes the development of a novel telemedicine-based ASD assessment tool, the TELE-ASD-PEDS-Preschool (TAP-Preschool). | Funded by the NIH/NIMH (R21MH118539), the Eunice Kennedy Shriver National Institute of Child Health and Human Development (U54 HD08321), and the Vanderbilt Institute for Clinical and Translational Research. |
| Wagner et al. | 2023b | United States | High income | Mixed Methods | The study aims to investigate the barriers to adopting patient-facing mobile applications for early intervention among underrepresented populations. | Funded by the Children and Youth with Special Healthcare Needs Network (UA6M31101) and the Learning Health Systems Scholars grant (K12 HS026395) from the Agency for Healthcare Research and Quality (AHRQ) and Patient-Centered Outcomes Research Institute (PCORI). |
| Warren et al. | 2013 | United States | High income | Qualitative | The study aims to examine the disparity in participation of lower-income African American smokers in internet-delivered smoking cessation interventions through the development and usability evaluation of an identity-congruent user interface. | Funded by the Prevent Cancer Foundation. |
| Wen et al. | 2014 | United States | High income | Qualitative | The study aims to describe the development of a social**-**cognitive theory-based and evidence-guided text messaging intervention for preventing postpartum smoking relapse among underserved women. | Funded by the National Institute of Health, American Cancer Society, Fox Chase Cancer Center Behavioral Research Core Facility. |
| Yee et al. | 2020 | United States | High income | Qualitative | The study aims to develop and pilot test a theory-driven curriculum of SMS text messaging for diabetes support and education during pregnancy. | Funded by the Evergreen Invitational Women’s Health Grants Initiative. |
| Yingling et al. | 2016 | United States | High income | Mixed Methods | The study aims to evaluate the feasibility and acceptability of physical activity (PA)-monitoring wristbands and Web-based technology by predominantly African American, church-based populations in resource-limited Washington, D.C. neighborhoods. | Funded by the National Institute of Health, Vignet Corporation. |
| You et al. | 2020 | South Africa | Upper middle income | Qualitative | The study aims to explore facilitators and barriers to incorporating mobile phones and advanced digital technologies (e.g., biometric identification, mobile phone apps, and chatbots) into HIV care for cisgender female sex workers living with HIV in Durban, South Africa. | Funded by the National Institute of Nursing Research of the National Institutes of Health (Award Number R01NR016650) and the Johns Hopkins University Center for AIDS Research (Award Number P30AI094189). |
| Zaim et al. | 2021 | United States | High income | Qualitative | The study aims to co-design with teen girls and parents, an internet-based training tool to teach teen girls the best way to find, evaluate, and use online health information. | Funded by a philanthropic donation from the Thirty-One Gives Foundation. |
| Zapata et al. | 2023 | United States | High income | Qualitative | The study aims to explore the preferred characteristics and willingness to adopt mobile health (mHealth) interventions among young sexual minoritized men aged 17-24 years in the United States, particularly related to HIV testing and prevention. | Funded by the National Institute of Mental Health (K01-MH118939, principal investigator: SAJ). The first authors’ time was supported by a training grant from the National Institute of Mental Health (T32MH130325; principal investigator: Newcomb). |
| Zingg et al. | 2022 | United States | High income | Mixed Methods | The study aims to outline an integrative content engineering framework to supplement existing design and development processes of peripartum depression digital solutions. | Not provided. |
