# Supplementary material for: Centering Equity During Health Technology Innovation: Scoping Review of Methods and Research Adjustments to Promote Inclusive Coproduction
Source: J Med Internet Res. 2026 Jul 3;28:e89596. doi: 10.2196/89596 (PMC13334495; doi:10.2196/89596)
Supplement: Multimedia Appendix 4 [file jmir-v28-e89596-s004.doc]

# Multimedia Appendix 04 Overlapping Priority Population Groups

| **Author** | **Year** | **Migrants and refugees** | **Women, girls, and pregnant women/people** | **People in vulnerable locations (including those in rural/remote settings and medically underserved settings)** | **People with disabilities (including mental health related) and their caregivers** | | **People living with HIV/AIDS** | **Older adults and their caregivers** | **Indigenous peoples** | **People marginalized by race/ethnicity** | **People marginalised by sexual orientation** | **People marginalised by gender identity (trans and gender diverse people)** | **Children and adolescents** | **People with low education/literacy levels** | **People of lower socioeconomic status** | **Language minority populations** | **People experiencing homelessness** | **People marginalised by occupation (sex work)** |
| --- | --- | --- | --- | --- | --- | --- | --- | --- | --- | --- | --- | --- | --- | --- | --- | --- | --- | --- |
| Aladin | 2023 |  |  |  | |  | x |  |  |  |  |  |  |  |  |  |  |  |
| Albright | 2015 |  |  | x |  | |  |  |  |  |  |  |  |  |  |  |  |  |
| Almond | 2016 |  |  | x |  | |  |  |  |  |  |  |  |  |  |  |  |  |
| Almond | 2017 |  |  | x |  | |  |  |  |  |  |  |  |  |  |  |  |  |
| Antonelli | 2021 |  |  |  | x | |  |  |  |  |  |  |  |  |  |  |  |  |
| Aronoff-Spencer | 2022 |  |  | x |  | |  |  |  |  |  |  |  |  |  |  |  |  |
| Baik | 2023 |  | x |  |  | |  |  |  | x |  |  |  |  |  |  |  |  |
| Bauer | 2018 |  |  |  | x | |  |  |  |  |  |  |  |  |  |  |  |  |
| Bendixen | 2017 |  |  |  | x | |  |  |  |  |  |  | x |  |  |  |  |  |
| Blackwell | 2020 | x | x |  |  | |  |  |  | x |  |  |  |  |  |  |  |  |
| Bounds | 2023 |  |  |  |  | |  |  |  |  |  |  | x |  |  |  |  |  |
| Bravo | 2014 |  | x |  |  | |  |  |  | x |  |  |  |  |  | x |  |  |
| Brewer | 2019 |  |  |  |  | |  |  |  | x |  |  |  |  |  |  |  |  |
| Brooks | 2021 |  |  |  | x | |  |  |  |  |  |  | x |  |  |  |  |  |
| Buckingham | 2023 |  |  |  | x | |  |  |  |  |  |  |  |  |  |  |  |  |
| Burchert | 2018 | x |  |  |  | |  |  |  |  |  |  |  |  |  | x |  |  |
| Calderon | 2017 |  | x | x |  | |  |  |  |  |  |  |  |  | x |  |  |  |
| Campbell | 2017 |  |  | x |  | | x |  |  |  |  |  |  | x |  |  |  |  |
| Carolan-Olah | 2021 |  | x |  |  | |  |  |  | x |  |  |  |  | x |  |  |  |
| Castillo | 2022 | x | x |  |  | |  |  |  | x |  |  |  |  |  | x |  |  |
| Ceasar | 2019 |  | x |  |  | |  |  |  | x |  |  |  |  |  |  |  |  |
| Cerda Diez | 2019 |  |  |  |  | |  |  |  |  |  |  |  |  |  | x |  |  |
| Champoux | 2020 |  | x |  |  | |  |  |  | x |  |  |  |  | x |  |  |  |
| Chandler | 2023 |  | x |  |  | |  |  |  | x |  |  |  |  |  |  |  |  |
| Chee | 2017 |  | x |  |  | |  |  |  | x |  |  |  |  |  |  |  |  |
| Cheng | 2020 |  |  |  |  | |  |  |  |  |  |  |  | x | x |  |  |  |
| Dal Bello-Haas | 2014 |  |  | x | x | |  |  |  |  |  |  |  |  |  |  |  |  |
| Dang | 2023 |  |  |  | x | |  |  |  | x |  |  |  |  |  |  |  |  |
| Day | 2021 |  |  | x |  | |  |  |  |  |  |  |  |  |  |  |  |  |
| Day | 2023 |  |  | x |  | |  |  | x |  |  |  |  |  |  |  |  |  |
| Dobson | 2017 |  |  |  |  | |  |  | x | x |  |  |  |  |  |  |  |  |
| Doty | 2020 |  |  | x |  | |  |  |  | x |  |  |  |  |  |  |  |  |
| Enyioha | 2023 |  |  |  |  | |  |  |  | x |  |  |  |  |  |  |  |  |
| Fontil | 2016 |  |  |  |  | |  |  |  | x |  |  |  |  | x |  |  |  |
| Garvelink | 2020 |  |  |  |  | |  | x |  |  |  |  |  |  |  |  |  |  |
| Givoenco | 2021 |  |  |  |  | | x |  |  |  |  |  | x |  |  |  |  |  |
| Godleski | 2020 |  | x |  |  | |  |  |  |  |  |  |  |  | x |  |  |  |
| Gordon | 2016 |  | x |  |  | |  |  |  | x |  |  |  |  | x |  |  |  |
| Greenhalgh | 2015 |  |  |  | x | |  | x |  | x |  |  |  |  | x |  |  |  |
| Grewal | 2023 |  |  |  |  | | x |  |  |  |  |  |  |  |  |  |  |  |
| Ha | 2023 |  |  |  | x | |  |  |  |  |  |  |  |  |  |  |  |  |
| Handley | 2016 | x | x |  |  | |  |  |  | x |  |  |  |  |  |  |  |  |
| Harris | 2023 |  |  |  |  | |  |  |  | x |  |  |  |  |  |  |  |  |
| Hearn | 2022 |  |  | x |  | |  |  |  |  |  |  |  |  |  |  |  |  |
| Henson | 2023 |  | x |  |  | |  | x | x |  |  |  |  |  |  |  |  |  |
| Higa | 2021 |  |  | x |  | |  |  |  |  |  |  |  |  |  |  |  |  |
| Hoque | 2017 |  |  |  |  | |  | x |  |  |  |  |  |  |  |  |  |  |
| Howells | 2022 |  |  |  |  | |  |  |  |  |  |  |  |  |  |  | x |  |
| Hughes | 2018 |  |  | x |  | |  |  |  |  |  |  |  |  |  |  |  |  |
| Hutchings | 2022 |  |  |  |  | |  |  |  | x |  |  |  |  |  |  |  |  |
| Hynie | 2022 | x |  |  | x | |  |  |  |  |  |  |  |  |  | x |  |  |
| Jenness | 2022 |  |  |  | x | |  |  |  |  |  |  | x |  |  |  |  |  |
| Jiam | 2017 |  |  |  | x | |  |  |  |  |  |  | x |  |  |  |  |  |
| Kang | 2023 |  |  |  | x | |  |  |  |  |  |  | x |  |  |  |  |  |
| Kayastha | 2021 |  | x | x |  | |  |  |  |  |  |  |  | x |  |  |  |  |
| Kothari | 2020 |  | x |  |  | |  |  |  |  |  |  |  |  | x |  |  |  |
| Lindegaard | 2022 |  |  |  |  | |  |  |  | x |  |  | x |  |  | x |  |  |
| Liu | 2019 |  |  |  | x | |  |  |  |  |  |  |  |  |  |  |  |  |
| Luo | 2021 |  |  |  |  | |  |  |  | x |  |  |  |  | x |  |  |  |
| Mafalda | 2020 |  |  |  | x | |  | x |  |  |  |  |  |  |  |  |  |  |
| Maragh-Bass | 2022 |  |  |  |  | |  |  |  | x |  |  |  |  |  |  |  |  |
| Mauka | 2021 |  |  |  |  | |  |  |  |  | x |  |  |  |  |  |  | x |
| Mayberry | 2016 |  |  |  |  | |  |  |  | x |  |  |  |  | x |  |  |  |
| McCall | 2021 |  | x |  |  | |  |  |  | x |  |  |  |  |  |  |  |  |
| McCall | 2022 |  | x |  | x | |  |  |  | x |  |  |  |  |  |  |  |  |
| Meijer | 2021 |  |  |  |  | |  |  |  |  |  |  |  |  | x |  |  |  |
| Merculieff | 2021 |  |  | x |  | |  |  | x |  |  |  |  |  |  |  |  |  |
| Miah | 2017 |  |  | x |  | |  |  |  |  |  |  |  |  |  |  |  |  |
| Morrow | 2017 |  |  |  |  | |  | x |  |  |  |  |  | x |  |  |  |  |
| Mueller | 2020 |  |  | x |  | |  |  |  |  |  |  |  |  |  |  |  |  |
| Nouri | 2019 |  |  |  | x | |  |  |  |  |  |  |  |  | x |  |  |  |
| Ospina-Pinillos | 2019 |  |  |  |  | |  |  |  |  |  |  | x |  |  | x |  |  |
| Owens | 2020 |  |  |  |  | |  |  |  | x |  |  |  |  |  |  |  |  |
| Pathak | 2021 |  |  |  | x | |  |  |  |  |  |  |  |  | x |  |  |  |
| Peng | 2022 |  |  |  |  | |  |  |  |  | x |  |  |  |  |  |  |  |
| Petros De Guex | 2023 |  |  |  |  | | x |  |  | x |  |  |  |  |  | x |  |  |
| Pipicella | 2023 |  |  |  |  | |  |  |  |  |  |  | x |  |  |  |  |  |
| Pluye | 2020 |  | x |  |  | |  |  |  |  |  |  |  | x | x |  |  |  |
| Povey | 2020 |  |  | x |  | |  |  | x |  |  |  | x |  |  |  |  |  |
| Radcliffe | 2021 |  |  |  | x | |  |  |  |  |  |  |  |  |  |  |  |  |
| Resnick | 2022 |  |  |  |  | |  |  |  | x |  |  |  |  |  |  |  |  |
| Robbins | 2019 |  |  |  |  | |  |  |  | x |  |  |  |  |  |  |  |  |
| Rozbroj | 2015 |  |  |  |  | |  |  |  |  | x |  |  |  |  |  |  |  |
| Russ | 2021 |  |  |  | x | |  |  |  | x | x |  |  |  |  |  |  |  |
| Shrestha | 2023 |  |  |  |  | |  |  |  |  | x |  |  |  |  |  |  |  |
| Simons | 2018 |  |  |  |  | |  |  |  |  |  |  |  | x |  |  |  |  |
| Spanhel | 2019 | x |  |  |  | |  |  |  |  |  |  |  |  |  |  |  |  |
| Sun | 2020 |  | x |  |  | |  |  |  |  |  | x |  |  |  |  |  |  |
| Swallow | 2016 |  |  |  |  | |  |  |  |  |  |  | x |  |  |  |  |  |
| Tonkin | 2017 |  |  | x |  | |  |  | x |  |  |  |  |  |  |  |  |  |
| Tremblay | 2021 |  |  |  | x | |  | x |  |  |  |  |  |  |  |  |  |  |
| van den Bergh | 2023 |  |  |  | x | |  |  |  |  |  |  |  |  |  |  |  |  |
| Van Dooren | 2023 |  |  |  | x | |  |  |  |  |  |  | x |  |  |  |  |  |
| Vangeepuram | 2018 |  |  |  |  | |  |  |  | x |  |  |  |  | x |  |  |  |
| Velez | 2014 |  |  | x |  | |  |  |  |  |  |  |  |  |  |  |  |  |
| Verbiest | 2019 |  |  |  |  | |  |  | x |  |  |  |  |  |  |  |  |  |
| Wagner | 2023a |  |  |  | x | |  |  |  | x |  |  |  |  |  | x |  |  |
| Wagner | 2023b |  |  | x | x | |  |  |  | x |  |  |  |  |  |  |  |  |
| Warren | 2013 |  |  |  |  | |  |  |  | x |  |  |  |  | x |  |  |  |
| Wen | 2014 |  | x |  |  | |  |  |  |  |  |  |  |  | x |  |  |  |
| Yee | 2020 |  | x |  |  | |  |  |  | x |  |  |  |  | x |  |  |  |
| Yingling | 2016 |  |  | x |  | |  |  |  | x |  |  |  |  | x |  |  |  |
| You | 2020 |  | x |  |  | | x |  |  |  |  |  |  |  |  |  |  | x |
| Zaim | 2021 |  | x |  |  | |  |  |  | x |  |  | x |  |  |  |  |  |
| Zapata | 2023 |  |  |  |  | |  |  |  |  | x | x |  |  |  |  |  |  |
| Zingg | 2022 |  | x |  | x | |  |  |  |  |  |  |  |  | x |  |  |  |
