# Supplementary material for: Centering Equity During Health Technology Innovation: Scoping Review of Methods and Research Adjustments to Promote Inclusive Coproduction
Source: J Med Internet Res. 2026 Jul 3;28:e89596. doi: 10.2196/89596 (PMC13334495; doi:10.2196/89596)
Supplement: Multimedia Appendix 5 [file jmir-v28-e89596-s005.doc]

# Multimedia Appendix 05 Digital Health Technology Classifications

Classification Categories

*Technology Type*

- Mobile Applications (Apps): Smartphone/tablet applications
- Web-based Platforms: Browser-based websites and portals
- Messaging Systems: Short message service platforms, text-based interventions, Unstructured Supplementary Service Data
- Telehealth/Virtual Care: Real-time remote consultation platforms
- Wearable/IoT: Wearable devices and Internet of Things integration
- Social media: Social networking platforms
- Automated Phone Systems: Interactive voice response systems

*Engagement Mechanism / Tasks*

- No standardisation

*User Engagement Model*

- Self-directed: Patient-initiated, autonomous engagement
- Self-directed (carer): Carer-initiated, autonomous engagement
- Self-directed (patient and carer) Both patient or carer-initiated, autonomous engagement
- Provider-mediated: Healthcare professional involvement required
- Guided/Coached: Professional support with regular check-ins (non-health professional)
- Automated/Push: System-initiated reminders and notifications
- Carer-supported: Support provided by carer
- Peer-supported: Community-based interaction and support

*Primary Health Function*

- Health Information / Education: Health information, education and health literacy
- Assessment/Screening: Health assessment, diagnostic and screening support
- Disease Management: Management of a serious medical condition
- Psychological Support / Wellbeing: Mental health support
- Behaviour Change: Initiates behaviour change and habit formation
- Self-monitoring: Data is captured by the patient / carer for future treatment and care
- Communication: Communication between people or health systems
- Care Coordination: Healthcare navigation and service coordination

*Clinical Condition / Health Domain*

- No standardisation

NOTE: Some may say ‘not specified.’ This may be due to the study being in the contextual inquiry or value specification stage, therefore, the study looks at digital health technologies more broadly. Additionally, the study may not have provided a specific description to be able to categorise the technology (e.g., mHealth, or digital health technology).

| **Author** | **Technology Platform** | **Engagement Mechanism / Tasks** | **User Engagement Model** | **Primary Function** | **Clinical Condition / Health Domain** |
| --- | --- | --- | --- | --- | --- |
| Aladin 2023 | Mobile App | Digital comic | Peer-supported | Disease Management | HIV |
| Albright 2015 | Messaging Systems | Information, resources, and activities | Automated/ Pushed | Behaviour Change | Cardiovascular health |
| Almond 2016 | Web-based Platform | Personal health record | Self-directed | Communication | Other complex chronic conditions |
| Almond 2017 | Web-based Platform | Personal health record | Self-directed | Communication | Other complex chronic conditions |
| Antonelli 2021 | Mobile App | Information, resources, and activities | Self-directed (patient and carer) | Health Information / Education | Visual impairments |
| Aronoff-Spencer 2022 | Mobile App | Electronic distress monitoring tool | Self-directed | Assessment/Screening | Cancer |
| Baik 2023 | Mobile App | Information, recommendations | Self-directed | Behaviour Change | Breast cancer |
| Bauer 2018 | Mobile App | Technology-mediated health service delivery (Psychology) | Provider-mediated | Psychological Support / Wellbeing | Mental health (posttraumatic stress and /or bipolar disorder) |
| Bendixen 2017 | Mobile App | Remote symptom monitoring | Self-directed (carer and patient) | Self-monitoring  Care Coordination | Brain and spinal cord anomalies |
| Blackwell 2020 | Messaging Systems | Information, resources | Automated/Pushed | Health Information / Education | Prenatal care |
| Bounds 2023 | Mobile App | Information | Self-directed | Psychological Support / Wellbeing | Adverse childhood experiences |
| Bravo 2014 | Mobile App | Questionnaire | Self-directed | Assessment/Screening | Breast cancer |
| Brewer 2019 | Mobile App | On-demand lifestyle intervention | Self-directed | Behaviour change support | Cardiovascular health |
| Brooks 2021 | Mobile App | Storyline, games and activities | Self-directed | Health Information / Education | Mental health (anxiety and depression) |
| Buckingham 2023 | Web-based Platform | Information, resources | Self-directed | Health Information / Education  Disease Management | Disability (movement impairment) |
| Burchert 2018 | Mobile App | Information, illustrations and weekly e-helper contact. | Self-directed Guided/Coached | Health Information / Education  Psychological Support / Wellbeing | Mental health (depression) |
| Calderon 2017 | Messaging Systems | Information, two-way communication | Self-directed (carer) | Health Information / Education  Communication | Common childhood illnesses |
| Campbell 2017 | Messaging Systems | Clinic attendance reminders | Automated/Push | Communication | HIV |
| Carolan-Olah 2021 | Web-based Platform | Education modules | Self-directed | Health Information / Education  Behaviour Change | Prenatal care |
| Castillo 2022 | Mobile App  Web-based Platform | Information, two-way communication | Provider-mediated | Assessment/Screening  Communication  Health Information / Education | Prenatal care |
| Ceasar 2019 | Mobile App | Motivational messages, education | Automated/Push | Behaviour Change | Physical activity |
| Cerda Diez 2019 | Web-based Platform | Conversational avatar | Self-directed | Assessment/Screening | Genetic history |
| Champoux 2020 | Messaging Systems | Healthy habits and self-monitoring prompts, Appointment scheduling | Automated/Push | Behaviour Change  Self-monitoring  Communication | Blood pressure |
| Chandler 2023 | Mobile App | Information, self-monitoring and health data logging, peer network | Self-directed  Peer-supported | Health Information / Education | Sexual health |
| Chee 2017 | Web-based Platform | Internet cancer support group, information, Care provider matching | Provider-mediated  Peer-supported | Disease Management  Care Coordination | Breast cancer |
| Cheng 2020 | Web-based Platform | Non-specific individual, family, practitioner and policy interventions proposed | Self-directed | Health Information / Education | General health support |
| Dal Bello-Haas 2014 | Telehealth/Virtual Care | Technology-mediated health service delivery | Provider-mediated  Carer-supported | Disease Management | Dementia |
| Dang 2023 | Web-based Platform | Animations, tips, and a virtual helper. | Self-directed (carer) | Health Information / Education | Dementia |
| Day 2021 | Telehealth/Virtual Care | Technology-mediated health service delivery | Provider-mediated | Psychological Support / Wellbeing | Mental health |
| Day 2023 | Telehealth/Virtual Care | Technology-mediated health service delivery | Provider-mediated | Psychological Support / Wellbeing | Mental health |
| Dobson 2017 | Messaging Systems | Information | Automated/Push | Health Information / Education | Parenting advice and nutritional information |
| Doty 2020 | Mobile App | Information | Self-directed | Health Information / Education | Parenting advice |
| Enyioha 2023 | Mobile App | Self-monitoring, tips | Self-directed | Behaviour Change  Self-monitoring | Smoking cessation |
| Fontil 2016 | Web-based Platform  Social media  Wearables/ IOT | Educational program, small group support, personalized health coaching, a weekly curriculum, and self-monitoring | Guided/Coached  Self-directed  Peer-support | Disease Management  Self-monitoring | Diabetes |
| Garvelink 2020 | Web-based Platform | Information | Self-directed (carers and patients) | Health Information / Education | Living independently |
| Givoenco 2021 | Mobile app  Web-based Platform | Information | Self-directed | Health Information / Education  Behaviour Change | HIV |
| Godleski 2020 | Mobile App | Information, resources, community forum | Self-directed  Peer-support | Health Information / Education  Behaviour Change | Mental health (parenting, and nutrition information) |
| Gordon 2016 | Web-based Platform | Actor and Animation guide, information, treatment options and selections, depression screening tool | Self-directed | Health Information / Education  Behaviour Change  Self-monitoring | Perinatal mental health |
| Greenhalgh 2015 | Telehealth/Virtual Care | Technology-mediated health service delivery | Provider-mediated | Communication  Disease Management | Multi-morbidity |
| Grewal 2023 | Telehealth/Virtual Care | Technology-mediated health service delivery | Provider-mediated | Disease Management | HIV |
| Ha 2023 | Mobile App  Web-based Platform  Wearables / IOT | Self-monitoring | Provider-mediated  Self-directed | Communication  Self-monitoring | Disability |
| Handley 2016 | Automated Phone Systems | Messages, education, tips, resources, narratives and self-monitoring prompts. | Guided/Coached  Self-directed | Health Information / Education  Disease Management  Behaviour Change  Self-monitoring | Gestational diabetes |
| Harris 2023 | Mobile App | Education, information, screening tools | Self-directed | Health Information / Education  Self-monitoring | Multi-morbidity |
| Hearn 2022 | Messaging Systems | Screening tool, triage tool, advice for patients and clinician alerts. | Provider-mediated  Self-directed | Disease Management  Self-monitoring | Cardiovascular disease |
| Henson 2023 | Not specified | Culturally sensitive, relevant information and illustrations | Not specified | Not specified | Older women |
| Higa 2021 | Telehealth/ Virtual Care  Wearables / IOT  Messaging Systems | Friends and family support, telehealth classes, personalized consultations, Bluetooth-enabled blood glucose monitors, and text messaging support. | Provider-mediated  Self-directed  Peer-support  Carer-supported | Health Information / Education  Disease Management  Behaviour Change  Self-monitoring | Diabetes |
| Hoque 2017 | Not specified | Not specified | Not specified | Not specified | Elderly people with chronic condition |
| Howells 2022 | Telehealth/ Virtual Care | Technology-mediated triage and health service delivery | Provider-mediated | Assessment/Screening  Disease Management | Covid-19 |
| Hughes 2018 | Mobile App | Information, training and assessment videos, appointment scheduling, two-way communication | Provider-mediated  Self-directed | Health Information / Education  Assessment/Screening  Disease Management  Communication | Stroke |
| Hutchings 2022 | Web-based Platform  Mobile App  Wearables/IoT | Information, two-way communication, self-monitoring | Provider-mediated  Self-directed | Assessment/Screening  Disease Management | Other complex chronic conditions |
| Hynie 2022 | Telehealth/Virtual Care | Phone, internet-based voice or video interactions, and text-based applications or messaging. | Provider-mediated | Psychological Support / Wellbeing | Mental health |
| Jenness 2022 | Mobile App | Chatbot, digital homework completion, logging moments and visualizations of mood and behaviour | Provider-mediated  Self-directed | Psychological Support / Wellbeing | Mental health |
| Jiam 2017 | Mobile App | Medical records organization | Self-directed (Carer) | Communication | Disability (Neurodevelopmental) |
| Kang 2023 | Mobile App | A picture-supported child-report instrument | Self-directed (Carer) | Assessment/Screening | Disability |
| Kayastha 2021 | Mobile App | Digital serious game designed for education, outreach and training purposes | Self-directed | Health Information / Education | Maternal and child health |
| Kothari 2020 | Mobile App | Daily note provided through push notification, videos, recipes and nutrition content, health information on mental health and child development, a forum for discussions, events and resources | Self-directed | Health Information / Education | Parenting advice and nutritional information |
| Lindegaard 2022 | Web-based Platform | Modules targeting various problem areas (including homework assignments), in-built messaging system to contact therapists, receive feedback on homework assignments and receive reminders | Guided/Coached | Psychological Support / Wellbeing | Mental health |
| Liu 2019 | Mobile App | Information, instructions, videos, interactive checklist to assess own wheelchair fit/set-up and skills, recommendations based on responses | Self-directed | Health Information / Education | Wheelchair use |
| Luo 2021 | Mobile App | Not specified | Self-directed | Disease Management | Diabetes |
| Mafalda 2020 | Web-based Platform | Information | Self-directed (carer) | Health Information / Education | Self-feeding |
| Maragh-Bass 2022 | Web-based Platform | Digital stories (combines video and audio) | Self-directed | Health Information / Education | Covid-19 |
| Mauka 2021 | Mobile App | Information on level of adherence, gamification, register pills taken, reminder messages, communication with trained peer educators and health care providers. Discussion forum, education materials, quizzes | Self-directed  Provider-mediated  Peer supported | Disease Management | HIV |
| Mayberry 2016 | Messaging Systems | Phone calls and text messaging | Guided/Coached | Disease Management | Diabetes |
| McCall 2021 | Mobile App | Thought journal, information, self-assessments, mood rating, graphs to track trends, self-care planner, links to resources | Self-directed | Psychological Support / Wellbeing  Self-monitoring | Mental health (anxiety & depression) |
| McCall 2022 | Mobile App | Thought journal, information, self-assessments, mood rating, graphs to track trends, self-care planner, links to resources | Self-directed | Psychological Support / Wellbeing  Self-monitoring | Mental health (anxiety & depression) |
| Meijer 2021 | Mobile App | Virtual coach which sends motivational messages, tips, information, personal stories, and videos for eight weeks through push notifications | Guided/Coached | Behaviour Change  Health Information / Education | Smoking cessation |
| Merculieff 2021 | Social Media | Information | Peer-supported | Behaviour Change  Health Information / Education | Smoking cessation |
| Miah 2017 | Mobile App | Allows GPs, based on queries and information support provided, to evaluate patient conditions virtually (comments, chat sessions, video calls) and provide answers for further diagnosis or treatment. Patients can rate GPs and medicine brands | Provider-mediated | Care Coordination | General health support |
| Morrow 2017 | Web-based Platform | Electronic medical record portal messages that convey test results | Provider-mediated | Communication | General health support |
| Mueller 2020 | Mobile App | Digital serious game | Self-directed | Health Information / Education | Maternal, neonatal health and geohazards |
| Nouri 2019 | Messaging Systems | Automated text messages | Automated/Push | Behaviour Change | Multi-morbidity |
| Ospina-Pinillos 2019 | Web-based Platform  Telehealth/Virtual Care | Triage system, self-report assessment, results dashboard, booking and videoconference system, generation of a personalized well-being plan | Provider-mediated | Care Coordination | Mental health |
| Owens 2020 | Mobile App | Audio-directed information and exercises | Self-directed (patient and carer) | Disease Management | Lung cancer |
| Pathak 2021 | Mobile App | Motivational messages | Self-directed | Behaviour Change | Physical activity |
| Peng 2022 | Mobile App | AI chatbot | Self-directed | Behaviour Change  Health Information / Education | HIV |
| Petros De Guex 2023 | Mobile App | Daily tracking of medication adherence, mood, and stress; appointment reminders; educational resources; laboratory results; and secure messaging with clinic staff and providers | Self-directed  Provider-mediated  Automated/Push | Disease Management  Self-monitoring  Communication | HIV |
| Pipicella 2023 | Web-based Platform | Cloud-based electronic clinical management system where clinicians perform clinical assessments, build management plans, and consumers complete questionnaires and report symptoms | Provider-mediated | Disease Management | Crohn's and colitis care |
| Pluye 2020 | Web-based Platform | Information with corresponding videos podcasts, and computer-audio-assistant highlighting sentences read | Self-directed | Health Information / Education | Early childhood development |
| Povey 2020 | Mobile App | Not specified | Self-directed | Health Information / Education | Mental health |
| Radcliffe 2021 | Mobile App | Logging meals, physical activity, and diet or fitness goals | Self-directed | Disease Management | Disability (weight management) |
| Resnick 2022 | Mobile App | Goal setting, tracking and sharing | Self-directed  Peer-supported | Self-monitoring | Cancer prevention |
| Robbins 2019 | Web-based Platform | Tailored messages and video narratives | Self-directed | Health Information / Education | Obstructive sleep apnoea |
| Rozbroj 2015 | Web-based Platform | Structured therapeutic program comprising reading, exercises, and evaluative tools | Self-directed | Health Information / Education | Mental health (anxiety & depression) |
| Russ 2021 | Mobile App | Information | Self-directed (patients and carers) | Health Information / Education | Surgical care |
| Shrestha 2023 | Mobile App | Clinic integrated, offers scheduling and managing appointments, communicating with the clinical team (ie, chat), home-based testing, accessing test results, ordering health products, discrete door-to-door delivery, timely notifications, a points-based reward system for completing activities within the app, and a multimedia resource centre. | Provider-mediated | Health Information / Education  Behaviour change  Communication  Care Coordination | HIV |
| Simons 2018 | Wearable/IoT  Mobile App | Information, goal setting, motivational notifications. Works in combination with a wearable activity tracker, the Fitbit Charge. The Fitbit Charge is a wrist-worn activity tracker that uses a 3-axis accelerometer to track a person’s movement. Active transport behaviour is being tracked by mobile smartphone sensors (GPS and accelerometer) | Self-directed Automated/Push | Behaviour Change  Health Information / Education  Self-monitoring | Physical activity |
| Spanhel 2019 | Web-based Platform | Information modules which include text and multimedia components (images, audios, videos), as well as reports from role models and interactive elements such as quizzes, a sleeping diary, and homework | Self-directed | Psychological Support / Wellbeing | Insomnia |
| Sun 2020 | Mobile App | A vision board, an education activity, and an interactive map which displays providers/organizations in five service categories: medical, support, educational, employment, and housing/living | Self-directed | Health Information / Education  Care Coordination | HIV |
| Swallow 2016 | Web-based Platform | Clinical support for care-giving (information on treatment regimens, video-learning tools, condition-specific cartoons/puzzles, and a question and answer area); and (2) psychosocial support for care-giving (social-networking, case-studies, or testimonials, managing stress and enhancing families’ health-care experiences) | Self-directed (carers) | Health Information / Education | Parents of children with chronic kidney disease (CKD) |
| Tonkin 2017 | Mobile App | Record consumption, demonstrates progress/feedback, games | Self-directed | Behaviour Change | Sugar sweetened beverage consumption |
| Tremblay 2021 | Web-based Platform | Search tool and a questionnaire to help identify the needs of caregivers, allows service providers to offer their services | Self-directed (Carers)  Provider-mediated | Care Coordination | Caring for older people |
| Van den Bergh 2023 | Wearable/IoT  Mobile App | Remote monitoring system (including a sensor necklace) which allows continuous tracking of physical activity and falls in daily life | Provider-mediated | Disease Management | Parkinson’s disease |
| Van Dooren 2023 | Mobile App | Interactive puzzle, information, choose own colour/avatar | Self-directed (patients and carers) | Communication | 22q11 deletion syndrome |
| Vangeepuram 2018 | Mobile App | Not specified | Self-directed | Health Information / Education | Physical activity |
| Velez 2014 | Mobile App | Collect form-based data that can be transmitted to an electronic health record. Data entry forms can be designed to provide point-of-care decision support and in-context health information | Provider-mediated | Care Coordination | General health support |
| Verbiest 2019 | Mobile App | Information, behavioural goal setting, user can invite others to join the challenge or task | Self-directed  Peer-supported | Behaviour Change | Physical activity |
| Wagner 2023a | Telehealth/Virtual care | A play-based instrument with assessment tasks and scoring procedures based on the child’s language | Provider-mediated | Assessment/Screening | Autism Spectrum Disorder |
| Wagner 2023b | Mobile App | Not specified | Self-directed | Care Coordination | Developmental delays |
| Warren 2013 | Web-based Platform | Information, discussion board, profile page | Self-directed  Peer-supported | Behaviour Change | Smoking cessation |
| Wen 2014 | Messaging Systems | Text messages, crave and lapse use-initiated functions | Automated | Behaviour Change | Smoking cessation |
| Yee 2020 | Messaging Systems | Text messages delivered via a web-based one-way messaging system | Automated | Disease Management  Health Information / Education | Gestational diabetes |
| Yingling 2016 | Wearable/IoT  Web-based Platform | Physical activity monitoring wristbands, centralised hub for capturing and transmitting data, internet account for tracking data | Self-directed | Behaviour Change | Physical activity |
| You 2020 | Not specified | Not specified | Not specified | Disease Management | HIV |
| Zaim 2021 | Web-based Platform | Webpage, information, videos, interactive game | Self-directed | Health Information / Education | General health support |
| Zapata 2023 | Mobile App | Not specified | Self-directed | Health Information / Education  Behaviour Change | HIV |
| Zingg 2022 | Web-based Platform | Not specified | Self-directed | Psychological Support / Wellbeing | Mental health (peripartum depression) |
