# Supplementary material for: Centering Equity During Health Technology Innovation: Scoping Review of Methods and Research Adjustments to Promote Inclusive Coproduction
Source: J Med Internet Res. 2026 Jul 3;28:e89596. doi: 10.2196/89596 (PMC13334495; doi:10.2196/89596)
Supplement: Multimedia Appendix 6 [file jmir-v28-e89596-s006.doc]

# Multimedia Appendix 06 List of Methods to Co-Produce Digital Health Technology at each Stage of CeHRes Roadmap

| **Methods** | **Description** | **References** |
| --- | --- | --- |
| **Contextual Inquiry (number of methods = 18, number of unique papers = 33)** | | |
| Body mapping | Body mapping typically involves participants tracing a life-sized outline of their own body (or using a pre-drawn body outline). Participants can then use drawing, painting, symbols, and text to represent physical sensations, emotions, trauma, experiences of care, and social or environmental influences on their health. Participants may then reflect on or interpret the visualisations individually or in a group setting. | (Povey, 2020) |
| Community advisory group | Community advisory groups (CAGs) are structured bodies composed of diverse community members who represent the interests and perspectives of a specific population. CAGs help identify real-world problems, priorities, and unmet needs in the community. They ensure the technology addresses relevant issues, avoiding misalignment between developer assumptions and user realities. CAGs provide ongoing guidance, feedback, and support throughout the co-development of a project. | (Aronoff-Spencer, 2022)  (Dobson, 2017)  (Hynie, 2022)  (Povey, 2020)  (Vangeepuram, 2018) |
| Cultural probes | Cultural probes include the use of cameras, diaries, and scrapbooks to map health care, social care, and socio-cultural needs of population. | (Greenhalgh, 2015) |
| eHealth Literacy questionnaire | An eHealth literacy scale is a validated tool used to measure an individual's ability to seek, find, understand, evaluate, and apply health information from electronic sources to address or solve a health problem. It helps assess how comfortable and capable people are in using digital tools manage their health. | (Cheng, 2020) |
| eHealth Literacy Scale (eHEALS) | The eHealth Literacy Scale (eHEALS), an 8-item self-report questionnaire. It measures perceived skills and confidence across areas such as knowing what health resources are available on the internet, how to use the internet to find helpful health information, and confidence in using online information to make health decisions. | (Zaim, 2021) |
| Focus groups | Focus groups typically include 6–10 participants from a target community and are guided by a trained facilitator. These sessions allow for a rich, collective exploration of experiences and perspectives. | (Blackwell, 2020)  (Dobson, 2017)  (Harris, 2023)  (Mueller, 2020)  (Peng, 2022)  (Russ, 2021)  (Simons, 2018)  (Verbiest, 2019)  (Wagner, 2023b)  (Wagner, 2023a)  (You, 2020)  (Zaim, 2021)  (Zapata, 2023)  (Zingg, 2022) |
| Home tour | Home tours involve the participants taking the researcher through their home, describing each room’s significance and activities that occur in it. | (Greenhalgh, 2015) |
| Interviews | Interviews are one-on-one conversations (structured, semi-structured, or unstructured) between a participant and a researcher or designer. | (Aronoff-Spencer, 2022)  (Blackwell, 2020)  (Bravo, 2014)  (Burchert, 2018)  (Castillo, 2022)  (Cheng, 2020)  (Dal Bello-Has, 2014)  (Doty, 2020)  (Greenhalgh, 2015)  (Hynie, 2022)  (Luo, 2021)  (Mafalda, 2020)  (Robbins, 2019)  (Tonkin, 2017)  (Wen, 2014)  (Yee, 2020)  (Zingg, 2022) |
| Photovoice and video methods | Photovoice is a method where participants capture photos (and sometimes captions or narratives) that represent aspects of their daily lives, environments, health behaviours, or barriers to care. These photos are then used to spark discussion and reflection, often in workshops or group settings. | (Povey, 2020) |
| Survey/questionnaire measuring Smartphone use | A smartphone use questionnaire can help researchers assess digital readiness and identify usage patterns. | (Tonkin, 2017) |
| Survey/questionnaire measuring older persons attitudes toward physical activity and exercise | Helps identify older adults’ perceptions, motivations, barriers, and facilitators related to physical activity and exercise. These insights are crucial for digital health, as they:   - Reveal emotional, cognitive, and social factors that influence engagement. - Help categorise users by readiness for behaviour change.   Allow developers to tailor features, tone, and user journeys in the digital tool to better fit user attitudes. | (Dal Bello-Has, 2014) |
| Survey/questionnaire measuring technology access, intention to use, tech attitudes and tech barriers (developed for study) | Survey designed by research team for study. Included questions focusing on:   - Measured access to mobile technology - Intention to use app - Technology attitudes   Barriers to technology use | (Doty, 2020) |
| Survey/questionnaire measuring health literacy, family involvement, adherence to selfcare, and comfort with cell phones (developed for study) | Survey designed by research team for study. Included questions focusing on:   - Health literacy - Family involvement - Adherence to self-care behaviours   Comfort using cell phone and text messaging | (Mayberry, 2016) |
| Survey/questionnaire measuring tech use, knowledge of e-mental health programs, and characteristics affecting acceptability of e-mental health resources (co-developed for study) | Survey collaboratively designed by research team and participants for the study. The 41-item online survey included questions focusing on:   - Current use of technology - Knowledge of available e-mental health programs   Characteristics which affect acceptability of e-mental health resources | (Povey, 2020) |
| Survey/questionnaire measuring technology use, smartphone ownership, tech attitudes, and potential app functions (co-developed for study) | Survey collaboratively designed by research team and participants for the study. The 26-item survey included questions focusing on:   - Technology use - Cell phone / smartphone ownership - Access to and usage of the internet - Use of social media, websites, and applications - Use of email/text messaging - Attitudes towards mobile technology - Internet and app use to obtain health information and track personal health information - Use of social media for community change   Potential app function | (Vangeepuram, 2018) |
| Think-aloud shop | A "think-aloud shop" is a research method where participants verbalise their thoughts and decision-making processes while shopping or selecting items. | (Tonkin, 2017) |
| Vignettes / scenario techniques | Vignettes are brief narratives or stories that illustrate a particular context, character, or challenge related to the use of digital health technology. They can be presented in written form, as videos, or as interactive scenarios, and are often followed by questions or discussion prompts. Vignettes shift the focus from personal disclosure to a third-person perspective, which helps reduce discomfort or defensiveness. | (Cheng, 2020)  (Povey, 2020) |
| Yarning | Yarning methodology is an Indigenous research method that involves conversational, story-based dialogue. It can be both one-on-one and in a group. | (Henson, 2023) |
| **Value Specification (number of methods n=16, number of papers n=43)** | | |
| Bus stop activity | Users are asked to engage with different mHealth tools for 5 minutes at each “bus stop.” Likes and dislikes are discussed within the group. | (Verbiest, 2019) |
| Card sorting | Card sorting involves participants organising topics, content, or features into categories that make sense to them. The cards typically represent individual pieces of information, actions, or functionalities (such as features, sections, or tools) that might appear in the final product. Participants sort these cards into groups, either based on predefined categories or by creating their own. This exercise helps reveal users’ preferences for organising content or functions. | (Greenhalgh, 2015) |
| Community advisory groups | See description under *Contextual Inquiry*. | (Antonelli, 2021)  (Brooks, 2021)  (Hynie, 2022) |
| End-user sketching / mock-ups | End-user sketching involves inviting users to draw their ideas, expectations, or visions for a tool.  Mock-ups are low- to medium-fidelity prototypes, usually paper-based or digital representations of a product’s interface or workflow. They are more structured than sketches and can be refined over time.  When used together, these tools enable a collaborative, iterative process where users can express their ideas through sketching, this can then be translated into mock-ups and then brought back to the community for feedback. | (Ospina-Pinillos, 2019) |
| Feedback on paper prototyping | Paper prototyping is a simple, low-fidelity way of creating visual representations of digital interfaces using paper, markers, and other physical materials. It allows users to interact with a tangible mock-up of the system, which can be iterated quickly based on feedback. This method involves the researcher using trigger materials (e.g., images showing features and capabilities of health-related apps) to encourage participants to build an app prototype. Paper prototypes allow for interactive discussions about which features or services would be most useful to them. For example, users can suggest which navigation paths feel most intuitive or what kinds of information they find most valuable. | (Tonkin, 2017)  (Verbiest, 2019) |
| Focus Groups | See description under *Contextual Inquiry*. | (Aladin, 2023)  (Albright, 2015)  (Antonelli, 2021)  (Bendixen, 2017)  (Brewer, 2019)  (Brooks, 2021)  (Buckingham, 2023)  (Godleski, 2020)  (Handley, 2016)  (Harris, 2023)  (Kothari, 2020)  (McCall, 2022)  (Peng, 2022)  (Rozbroj, 2015)  (Wagner, 2023b)  (Simons, 2018)  (Sun, 2020)  (Verbiest, 2019)  (Wagner, 2023a)  (Warren, 2013)  (Zaim, 2021)  (Zapata, 2023) |
| Information Assessment Method Questionnaire | The Information Assessment Method (IAM) Questionnaire is a tool used to assess an individual’s ability to find, understand, and use information effectively, particularly in the context of health information. | (Pluye, 2020) |
| Interviews | See description under *Contextual Inquiry*. | (Aladin, 2023)  (Baik, 2023)  (Brooks, 2021)  (Calderon, 2017)  (Champoux, 2020)  (Doty, 2020)  (Enyioha, 2023)  (Hearn, 2022)  (Hynie, 2022)  (Merculieff, 2021)  (Mauka, 2021)  (Pluye, 2020)  (Swallow, 2016)  (Wen, 2014) |
| Member checking | Member checking involves sharing back materials, findings, or design outputs with community participants who contributed earlier in the co-development process and asking for their feedback, clarification, or endorsement. | (Povey, 2020) |
| Persona | A persona is a fictional character created to represent a particular segment of the target population. It is based on data gathered through research (e.g., surveys, interviews, observations) and reflects the typical behaviours, motivations, goals, and challenges of real users. | (Ha, 2023)  (Verbiest, 2019) |
| Survey/questionnaire measuring importance of potential information and design features of DHT (developed for the study) | Survey designed by research team for study. Included questions focusing on:   - Importance of information - Importance of design features | (Doty, 2020) |
| Use-cases | A use-case is a detailed, narrative description of how a specific user (or type of user) interacts with a digital health tool to accomplish a particular goal. Use-cases are typically written in plain language, often enriched with visuals or storyboards. | (Swallow, 2016) |
| Vignettes / scenario techniques | See description under *Contextual Inquiry*. | (Greenhalgh, 2015)  (Ha, 2023) |
| Voting rounds | Participants are asked to vote on potential functionalities to be included in the design of the DHT | (Pipicella, 2023) |
| Workshops | Members of a community come together to generate ideas, define values, and co-create solutions for digital health technology. Participants brainstorm or prototype features that embody the prioritised values. Groups present their ideas and receive feedback. This creates a shared understanding of which values should guide design and how they might be realized. | (Aronoff-Spencer, 2022)  (Dang, 2023) |
| Yarning | See description under *Contextual Inquiry*. | (Henson, 2023) |
| **Design (number of methods n=30, number of unique papers n=72)** | | |
| Asynchronous Remote Communities Methods | ARC involves forming online communities where participants interact and engage in structured discussions over an extended period (typically weeks), but asynchronously, meaning they contribute at times convenient to them. These communities are typically hosted on private digital platforms (e.g., Slack, Discord, private forums) moderated by researchers or facilitators. | (Bounds, 2023)  (Jenness, 2022) |
| Card sorting | See description under *Value Specification.* | (Nouri, 2019)  (Pathak, 2021) |
| Community advisory group | See description under *Contextual Inquiry*. | (Bauer, 2018)  (Ceasar, 2019)  (Maragh-Bass, 2022)  (Sun, 2020) |
| Content validity form | A content validity form is a tool used to evaluate the relevance and appropriateness of the content included in a digital health technology, ensuring it meets the needs and expectations of the target community. Participants may be invited to review the DHT and complete the form which may include items such as: (a) Rate overall topic (essential; important, but not essential; optional; not relevant); (b) Is content age-appropriate? (yes/no); (c) Is content accurate? (yes/no); and (d) Do you suggest any changes or additions? (yes/no). | (Antonelli, 2021) |
| Cultural context assessment | A meeting that focuses on positioning the proposed DHT into the cultural context of potential community and elicit feedback on culturally tailored features of the intervention. | (Brewer, 2019) |
| Cultural sensitivity assessment tool | Can utilise the Cultural Sensitivity Checklist (Friedman & Hoffman-Goetz, 2006) and Cultural Sensitivity Assessment Tool (Guidry & Walker, 1999) to inform one’s interview guide to help assess the cultural appropriateness of the DHT, ensuring it aligns with the cultural values, beliefs, and practices of specific communities. | (Owens, 2020) |
| Desirability matrix | A desirability matrix is a tool used to evaluate and prioritize features, functionalities, or design elements based on how much they are desired by the target users. Participants are given various adjectives to describe their experience of using the DHT. Some adjectives are unfavourable (e.g., unclear, distracting, hard  to read), others are favourable (e.g., trustworthy, clean, attractive). | (Warren, 2013) |
| Digital storytelling workshops | Digital storytelling workshops involve participants creating short, multimedia narratives—combining personal voice, images, video, and music—to tell their health-related stories. These stories often focus on illness journeys, care experiences, or barriers to accessing health services. | (Maragh-Bass, 2022) |
| DISCERN questionnaire | The DISCERN questionnaire is a standardized tool used to assess the quality of written health information, particularly regarding treatment choices. It evaluates content based on clarity, reliability, balance, and the extent to which it supports informed decision-making. | (Swallow, 2016) |
| Eye tracking glasses and software | Eye tracking glasses and software allow designers and researchers to monitor and analyse the visual attention of users as they interact with digital interfaces, offering insights into how users perceive, navigate, and engage with the technology. | (McCall, 2021)  (Warren, 2013) |
| Focus groups | See description under *Contextual Inquiry*. | (Aladin, 2023)  (Albright, 2015)  (Bounds, 2023)  (Burchert, 2018)  (Ceasar, 2019)  (Fontil, 2016)  (Givoenco, 2021)  (Godleski, 2020)  (Handley, 2016)  (Hearn, 2022)  (Jiam, 2017)  (Kayastha, 2021)  (Kothari, 2020)  (Liu, 2019)  (Mauka, 2021)  (Miah, 2017)  (Mueller, 2020)  (Russ, 2021)  (Wagner, 2023a)  (Yingling, 2016) |
| Health literacy advisor software | A Health Literacy Advisor Software (Health Literacy Innovations, LLC) is a tool used to assess and improve the readability of digital health content, ensuring that it is accessible, understandable, and actionable for a wide range of users, especially those from priority communities. | (Wen, 2014) |
| Internet evaluation and utility questionnaire (IEUQ) | The Internet Evaluation and Utility Questionnaire provides insight into how users perceive the quality, trustworthiness, and usefulness of health-related information found online. This questionnaire assesses key factors such as users’ ability to evaluate the credibility of online sources, their satisfaction with the information they find, and how helpful they perceive that information to be in managing their health. | (Carolan-Olah, 2021) |
| Interviews | See description under *Contextual Inquiry*. | (Aladin, 2023)  (Albright, 2015)  (Bravo, 2014)  (Cerda Diez, 2019)  (Chandler, 2023)  (Dobson, 2017)  (Garvelink, 2020)  (Ha, 2023)  (Higa, 2021)  (Lindegaard, 2022)  (Mayberry, 2016)  (Meijer, 2021)  (Morrow, 2017)  (Nouri, 2019)  (Owens, 2020)  (Pathak, 2021)  (Petros De Guex, 2023)  (Resnick, 2022)  (Shrestha, 2023)  (Swallow, 2016)  (Tonkin, 2017)  (Tremblay, 2021)  (van den Bergh, 2023)  (Van Dooren, 2023)  (Velez, 2014)  (Wagner, 2023a)  (Yee, 2020) |
| Member checking | See description under *Value Specification.* | (Dang, 2023) |
| Observational methods | Observational methods involve directly watching users, such as patients, clinicians, or caregivers, as they engage with the digital health technology in real-world environments. The goal is to understand actual behaviour, workflows, and context, rather than relying solely on self-reported data.  E.g., think-aloud, cognitive walkthroughs, ethnographic contextual observation | (Aronoff-Spencer, 2022)  (Bounds, 2023)  (Bravo, 2014)  (Buckingham, 2023)  (Burchert, 2018)  (Champoux, 2020)  (Doty, 2020)  (Hearn, 2022)  (Hughes, 2018)  (McCall, 2021)  (Ospina-Pinillos, 2019)  (Resnick, 2022)  (Robbins, 2019)  (Spanhel, 2019)  (Simons, 2018)  (Sun, 2020)  (Tonkin, 2017)  (Tremblay, 2021)  (Velez, 2014)  (Warren, 2013)  (Wen, 2014) |
| Participatory design meetings | Participants generate ideas and provide feedback on pilot software including favoured language, features, and stylistic elements of the “look” of the DHT that were appropriate to the target population. Additionally, participants provide input on literacy and readability challenges with language. | (Gordon, 2016) |
| Role-playing | Role-playing allows researchers to review the impact of the applications on clinical workflows; participants of the group can observe proposed workflows and provide immediate responses and suggestions for modifications which could then be discussed for issues such as technical feasibility. | (Gordon, 2016) |
| Satisfaction surveys | Satisfaction surveys typically ask users to rate their satisfaction with various aspects of the DHT—such as content relevance, ease of use, visual appeal, cultural appropriateness, and perceived usefulness. They help identify what is working well and where adjustments are needed to improve engagement, trust, and usability. | (McCall, 2021)  (Morrow, 2017)  (Simons, 2018)  (Swallow, 2016)  (Wagner, 2023a) |
| Simple Measure of Gobbledygook rating | The Simple Measure of Gobbledygook (SMOG) is a readability formula used to estimate the years of education a person needs to understand a piece of written content. The tool can help researchers ensure that written materials—such as health information, app instructions, or interface text—are accessible to users with varying levels of health literacy and formal education. | (Fontil, 2016)  (Swallow, 2016) |
| Suitability Assessment of Materials (SAM) measure | The Suitability Assessment of Materials (SAM) measure is a validated tool used to evaluate the appropriateness, accessibility, and cultural relevance of health education materials. | (Swallow, 2016) |
| Survey/questionnaire measuring clarity and adequacy of the information, relevance, design and functionality of DHT (developed for study) | Survey designed by research team for study based on a Likert scale. Evaluated the terminology used, the clarity and adequacy of the information, the relevance, design and functionality of the DHT. | (Mafalda, 2020) |
| Survey/questionnaire measuring perceived effectiveness | The perceived effectiveness survey is a 6-item validated measure capturing end-users' perceptions of how well a digital health intervention might work for their specific circumstances. Participants reported how much they agreed with six statements:   - This was worth remembering - This grabbed my attention - This was powerful - This was informative - This was meaningful   This was convincing | (Merculieff, 2021) |
| Survey/questionnaire measuring satisfaction, ease of use, clarity, perceived value, and barriers to use  (developed for study) | Survey designed by research team for study. Included questions focusing on:   - Perceptions and satisfaction with DHT - Ease of use - Clarity of instructions - Timing - Perceived value - Barriers to use   Appropriateness for specific needs | (Wagner, 2023b) |
| System Usability scale | The System Usability Scale (SUS) is a widely used tool for evaluating the usability of a system or digital product. SUS consists of a 10-item questionnaire that assesses users’ perceptions of a system’s ease of use, complexity, consistency, and overall satisfaction. | (Aronoff-Spencer, 2022)  (Bauer, 2018)  (Bounds, 2023)  (Chandler, 2023)  (Hughes, 2018)  (Petros De Guex, 2023)  (Radcliffe, 2021)  (Shrestha, 2023) |
| Tierney’s 7-min Accessibility Assessment and App Rating | Developed by Tierney et al. (2022), this tool provides a structured, time-efficient framework for assessing an app’s accessibility features in under 10 minutes. It focuses on evaluating critical elements such as:   - Text readability - Visual design (e.g., contrast, font size) - Navigation simplicity - Use of icons and symbols - Audio/visual alternatives - Compatibility with assistive technologies   The tool includes a rating system that scores the app across various domains of accessibility, and provides quick, actionable insights for developers and stakeholders. | (Radcliffe, 2021) |
| Usability surveys other than SUS | Usability surveys typically assess key dimensions such as ease of use, clarity of information, navigation, visual design, user satisfaction, and perceived effectiveness. | (Baik, 2023)  (Brewer, 2019)  (Jiam, 2017)  (Kang, 2023)  (Liu, 2019)  (Velez, 2014) |
| Usefulness survey | Usefulness surveys focus on users’ perceptions of how helpful the digital health technology is in managing their health—such as improving understanding of a condition, supporting decision-making, or facilitating communication with providers. | (Robbins, 2019) |
| Wireframes | Wireframes are low-fidelity, simplified representations of a digital interface (e.g., a mobile app, website, or kiosk). They outline layout, structure, and key functions without the distraction of final visuals, colours, or branding. | (Swallow, 2016)  (Verbiest, 2019) |
| 5 second test | A 5-second test involves showing participants a single screen or interface (usually a landing page, home screen, or key interaction point) for just 5 seconds, then asking them questions about what they saw and understood. | (Tremblay, 2021) |
| **Operationalization (number of methods n=6, number of unique papers n=14)** | | |
| Community advisory group | See description under *Contextual Inquiry*. | (Doty, 2020)  (Grewal, 2023) |
| Focus groups | See description under *Contextual Inquiry*. | (Almond, 2016)  (Russ, 2021)  (Wagner, 2023a) |
| Interviews | See description under *Contextual Inquiry*. | (Almond, 2016)  (Almond, 2017)  (Cambell, 2017)  (Dal Bello-Has, 2014)  (Day, 2021)  (Day, 2023)  (Fontil, 2016)  (Howells, 2022)  (Hutchings, 2022) |
| Logic modelling | A logic model serves as a visual framework that outlines the inputs, activities, outputs, outcomes, and impacts of a health intervention or program. The logic model can help to clarify goals, track progress, and ensure alignment between all stakeholders involved in the implementation of the technology. Additionally, a logic model allows people to test any assumptions they may have about who will or won’t use the technology and any barriers / facilitators to uptake. | (Hutchings, 2022) |
| Stakeholder mapping | Stakeholder mapping is a tool used to identify and understand the roles, interests, and influence of various individuals, groups, or organizations that are involved in or affected by a project. | (Hutchings, 2022) |
| Survey / Questionnaire measuring adoption facilitators and barriers | A questionnaire measuring adoption facilitators and barriers is a tool used to identify and assess the factors that either facilitate or hinder the adoption of a digital health technology within a specific community or user group. | (Hoque, 2017) |
| **Summative evaluation (number of methods n=2, number of unique papers n=2)** | | |
| Built-in mechanisms for gathering feedback | A feedback request serves as a structured method to gather input from users and community stakeholders once digital health technology has been implemented. By actively seeking feedback, developers can ensure that the community’s experiences and perspectives are integral to the evaluation process. This feedback informs whether the technology is meeting community needs, where adjustments might be necessary, and how the intervention might be improved or scaled. | (Buckingham, 2023) |
| Community advisory group | See description under *Contextual Inquiry*. | (Doty, 2020) |
