# Supplementary material for: Centering Equity During Health Technology Innovation: Scoping Review of Methods and Research Adjustments to Promote Inclusive Coproduction
Source: J Med Internet Res. 2026 Jul 3;28:e89596. doi: 10.2196/89596 (PMC13334495; doi:10.2196/89596)
Supplement: Multimedia Appendix 7 [file jmir-v28-e89596-s007.doc]

# Multimedia Appendix 07 Recommendations for Applying Scoping Review Findings

| **CeHRes Stage** | **CeHRes Sub-Stage** | **Recommendations for Engaging with Priority Populations** | **Tools identified** |
| --- | --- | --- | --- |
| **Contextual Inquiry** | **Stakeholder Identification:** | - Identify the priority populations and map based on their power, legitimacy, and urgency. - Establish a formal community governance by establishing a formal, funded community advisory board with decision-making authority to ensure community oversight and advice throughout the stages of development. - Co-develop a formal charter with the community advisory board that documents the project's commitment to equity, data sovereignty, cultural safety, and shared values. - Establish formal community recruitment, engage peer-led recruitment approaches and peer researchers (individuals with similar lived experience) to build trust and effectively reach community members. | - Body mapping - Cultural probes - eHealth literacy scale - eHealth literacy questionnaire - Home tour - Photovoice and video methods - Think aloud shop - Workshops - Surveys and questionnaires |
|  | **Current State Analysis** | - Understand the current situation, systematically map user behaviours, organisational workflows, current technology and wider policy context, to define the problem and who it affects. - Map access barriers and equity risks, to understand the lived experience of priority populations. Establish baseline access and capabilities such as access to devices, connectivity, digital skills, language and literacy, interpreter and accessibility needs. - Consider safety, privacy, data ownership, ethical issues, consent and anonymity risks, concerns, preferences, and expectations within the priority populations. |  |
|  | **Problem & Opportunity Identification:** | - Pinpoint specific points for improvement, for example improvements to inefficiency, safety, or satisfaction, and define the target behaviours that the technology aims to change. - Use a range of culturally grounded tools to allow the community participants to define the problem and opportunities in their own terms, respecting diverse ways of understanding and communicating. - Explore use of narrative methods such as Yarning to create a culturally safe space for patients to share their health stories or vignettes to define the problem from the community's perspective. - Ensure consideration to personal or spiritual beliefs by, for example, opening with a prayer or culturally relevant message, if appropriate. - Consider group dynamics by splitting groups by gender, age, or sexual orientation to create safe spaces for discussion. - Create an accessible & safe environment and ensure participant safety and comfort by co-developing ground rules and checking in on comfort levels. - Conduct all community engagement activities in trusted and accessible community-based locations (e.g., participant homes, community centres, religious communities), to reduce participation barriers (cost, travel, mistrust) and increase psychological safety for participants. - Use arts-based methods or play to engage children and young people - Conduct all activities in the priority population's language or engage a suitable interpreter. |  |
|  | **Stakeholder Engagement and Data Collection** | - Consider group dynamics by splitting groups by gender, age, or sexual orientation to create safe spaces for discussion. - Create an accessible & safe environment and ensure participant safety and comfort by co-developing ground rules and checking in on comfort levels. - Conduct all community engagement activities in trusted and accessible community-based locations (e.g., participant homes, community centres, religious communities), to reduce participation barriers (cost, travel, mistrust) and increase psychological safety for participants. - Use arts-based methods or play to engage children and young people - Conduct all activities in the priority population's language or engage a suitable interpreter. |  |
| **Value Specification** | **Value & Goal Formulation:** | - Elicit and prioritise the core values, such as social, behavioural, cognitive, emotional, economic, or health care issues, from all key stakeholders. - Translate these abstract values into concrete, SMART (specific, measurable, achievable, realistic, and timely) objectives. - Ensure this stage is community-led, where community participants are empowered to define and prioritise the core values and objectives of the project. - A combination of tools and approaches can be used to gather values, such as interviews, focus groups, or questionnaires, which should be tailored to the needs of the community. - Use visual and tactile methods for prioritisation activities that are accessible to all literacy levels. | - End user sketching and mock ups - Feedback on paper prototyping - Information assessment method questionnaire - Persona - Use cases - Voting rounds - Surveys and questionnaires |
|  | **Technology Requirements & Feature Selection:** | - Translate values and objectives into specific functional and non-functional requirements for the technology - Select evidence-based behaviour change techniques (BCTs) and persuasive features that align with stakeholder values. - Develop personas and use-case scenarios to guide the technology design, ensure personas are co-created and representative of each priority population group - Implement literacy & accessibility audits for all requirement-gathering activities - Ensure consideration is given to social determinants and technology barriers, which may include language, literacy, device and data access, trust factors, cultural protocols, safeguarding needs, consent preferences, and support networks - Provide oral instructions, visual aids, and non-digital alternatives to ensure that individuals with low literacy or digital skills can contribute equally to defining the technology's features and ensure full participation. |  |
|  | **Initial Implementation Planning:** | - Create a first version of a business model or logic model for example using a business model canvas, to plan for long-term sustainability and implementation. - Integrate an "Equity Impact Assessment" into the business model canvas and explicitly analyse the potential impact on priority populations, for example, potential financial and accessibility impact and establish a plan to mitigate these barriers and ensure the value proposition is genuinely beneficial for the breadth of diverse users. |  |
| **Design** | **Prototyping:** | - Establish a dedicated "Co-design Team" composed of community members, stakeholders and peer researchers to lead participatory design sessions - Develop both low-fidelity (e.g., paper-based sketches, wireframes) and high-fidelity (interactive) prototypes based on the defined requirements. Consider using arts-based methods, which may address language and literacy preferences. - Make use of tools like images and emotion cards to facilitate communication for those who may find verbal expression difficult. - Gather insights into barriers that might arise during implementation from priority populations, connecting development and implementation, record likely exclusion points and the adaptations required. - For all user-facing interactions, start sessions by co-creating the ground rules for safety and respect, and build in regular, private check-ins to monitor comfort levels. - Allow participants to bring a trusted support person, especially when dealing with sensitive health topics.   . | - Asynchronous remote communities method - Content validity form - Cultural context assessment - Cultural sensitivity assessment tool - DISCERN questionnaire - Desirability matrix - Eye tracking glasses and software - Health literacy advisor software - Observational methods - Participatory design meetings - Role playing - Satisfaction surveys - Simple measure of gobbledegook - Suitability assessment of materials - System usability scale and usability surveys - Internet evaluation and utility questionnaire - Tierney seven minute accessibility assessment - Wireframes - Five second test - Surveys and questionnaires |
|  | **Refinement of Approach:** | - Use participatory design and co-creation sessions with stakeholders to refine the prototypes and update in an iterative process - Integrate the selected BCTs and persuasive features into the prototype designs. |  |
|  | **Usability Testing & Evaluation:** | - Conduct iterative usability tests on the prototypes using representative intended users and experts using a range of appropriate tools such as the system usability scale, think-aloud method or heuristic evaluation. - Ensure the measurement of what matters to the population when capturing usability, satisfaction, and desirability. - To widen participation, provide for multiple, individualised ways for users to provide feedback (e.g., one-on-one sessions with a peer researcher, take-home kits, voice-note or phone feedback) to accommodate diverse needs, schedules, and comfort levels with technology and group settings, - Accommodate diverse contexts and allow for assisted or proxy use, paper or low data paths, small screen flows, and first language task scripts so priority groups can participate comfortably. - Protect privacy and safety through requiring pseudonyms for all participants during sessions and in reports; default to no recording unless consented; minimise data collected and confirm clear withdrawal routes. |  |
| **Operationalisation** | **Implementation Strategy and Planning:** | - Plan for the phased introduction, dissemination, adoption of a first functioning version of the technology in the intended context. - Designate trusted community-based organisations as implementation partners agree clear responsibilities for outreach, recruitment, training, support, and follow up; set budget, data sharing and safeguarding arrangements. Co-create the rollout strategy with them. - Allocate budget to fund the CBO’s role in training, support, and outreach, leveraging their existing community relationships and trust within local communities. - Include implementation support and inclusion enablers such as assisted and proxy use, in-language training or device support, interpreters, peer supporters and digital champion networks, helpline and community drop-ins, non-digital routes; define roles, costs, value, and simple success measures. - Select and test implementation strategies learn and adapt approach. - Finalise the business model with intended users, developing concrete plans on how to act upon the content of the 9 blocks of the business model canvas in close cooperation with stakeholders, using methods such as focus groups, desk research, or interviews - Synthesise barriers and facilitators by population that links each factor to a concrete mitigation strategy and record risks, owners, and timing. - Define outcomes and equity thresholds for example, acceptability, reach, engagement, adherence, cost to users and services, and sustainability; collect disaggregated data and review with community representatives. |  |
|  | **Implementation Execution:** | - Train and compensate peer supporters from the community to lead onboarding, provide culturally appropriate troubleshooting, and act as trusted champions for the technology. - Enable inclusive access to training, using trusted community-based venues, flexible timing, device loans and data vouchers, offline and low data modes. - Use peer support resources to provide one to one onboarding, small group sessions, drop-in clinics, a phone line, and voice note options. - Ensure all training materials are adapted for low literacy levels and are available in the priority population's language, adapted for low literacy levels and use of multiple channels, e.g. written in plain language, available as large print, audio, and short videos with materials, co-created with community organisations and stakeholders. - Run real time feedback and improvement sessions which collect blended feedback through paper, phone, and in app prompts. - Hold regular review huddles with community partners and update training and support materials quickly in response to feedback - Monitor equity during rollout, track reach, engagement, and adherence by population subgroup, share simple progress summaries back with communities. |  |
| **Summative Evaluation** | **Impact Assessment:** | - Determine the technology's ***impact*** on users and their context by evaluating clinical, behavioural, and organisational outcomes. - Mandate Equity as a primary outcome, designed to establish whether the DHT reduced, maintained, or widened health disparities. Pre-specify the stratification of key outcome data by relevant demographic variables (e.g., race, income, language). - Define outcomes and measures such as equity, acceptability, trust, comprehension, burden, and cost to users, reach, engagement, adherence, and fidelity; set subgroup targets and success thresholds. - Stratify the user population by relevant variables such as language, income, ethnicity, disability, age, and geography - Define questions which translate values into research questions that ask if, why, how, for whom, and when the technology works - Establish a range of data collection options that include paper, phone, and assisted completion. |  |
|  | **Uptake Analysis:** | - Analyse ***use***; how, when, and by whom the technology is used, primarily through the analysis of log data and built-in feedback mechanisms. - Disaggregate all log data to identify whether there are differences in uptake or feature use between priority population subgroups. - When disparities are found, make use of qualitative methods to understand "why”, through use of the established community groups and stakeholders. - Use strategies like daily phone calls or other regular check-ins to enhance engagement and minimise dropouts during the evaluation period. |  |
|  | **Impact Mechanism Investigation:** | - Investigate ***why*** the technology was or was not effective within the intended population by exploring and mapping the relationship between context, mechanisms of impact, such as selected behaviour change techniques, engagement and adherence, and outcomes, for whom and when. - Co-develop accessible, culturally appropriate materials (e.g., community meetings, videos) to share results and learnings back with the community population. - Establish a community-led process and involve community partners to analyse and interpret results; present findings in accessible formats and languages; capture lessons and feedback to inform the next iteration. - Triangulate quantitative data, disaggregated by priority subgroups, with qualitative data gathered using interviews, focus groups and observations that document unintended effects and burden on users. - Apply pre-established equity thresholds to inform decisions on whether to continue, adapt, or stop implementation. - Record specific design or implementation challenges and unintended effects and required adaptations or changes and update the logic model, and evaluation plan. |  |
